# Linking dietary patterns to Alzheimer's disease biomarkers with network mathematical modeling could enable new approach methodologies in preventative AD research: a narrative interdisciplinary review

**DOI:** 10.3389/fnut.2025.1673533

**Published:** 2026-01-12

**Authors:** Travis B. Thompson, Andrew C. Shin, Boris Decourt, Yifan Wang, Bradley Z. Vigil, Anna Solodukhina, Shakkya Ranasinghe, Robert S. Young, Vijay Hegde, Naima Moustaïd-Moussa

**Affiliations:** 1 Institute for One Health Innovation, Texas Tech University System, Lubbock, TX, United States; 2 Department of Mathematics and Statistics, Texas Tech University, Lubbock, TX, United States; 3 Center of Excellence for Translational Neuroscience and Therapeutics, Texas Tech University Health Sciences Center, Lubbock, TX, United States; 4 Center of Excellence in Obesity and Cardiometabolic Research, Texas Tech University and Texas Tech Health Sciences Center, Lubbock, TX, United States; 5 Human Molecular Aging Center, Texas Tech University, Lubbock, TX, United States; 6 Neurobiology of Nutrition Laboratory, Department of Nutritional Sciences, College of Health and Human Sciences, Texas Tech University, Lubbock, TX, United States; 7 Department of Pharmacology and Neuroscience, Texas Tech University Health Sciences Center, Lubbock, TX, United States; 8 Department of Nutritional Sciences, Texas Tech University, Lubbock, TX, United States; 9 Department of Cell Biology and Biochemistry, School of Medicine, Texas Tech University Health Sciences Center, Lubbock, TX, United States; 10 School of Veterinary Medicine, Texas Tech University, Amarillo, TX, United States

**Keywords:** Alzheimer's disease, diet, nutrition, mathematical modeling, network neurodegeneration

## Abstract

Alzheimer's disease (AD) is a significant global health concern. With no reliable pharmaceutical treatments on the horizon, the best path forward is preventative. Dietary patterns are related to one third of AD risk factors and have long been thought to influence the onset or the progression of AD. Studies of the preventative possibilities of diet on AD offer the prospect of helping to suppress AD prevalence until effective pharmaceutical interventions are discovered but can be challenging due to variations, duration, cost or ethical considerations presented by human and animal studies. At the same time, the National Institutes of Health and the Food and Drug Administration are encouraging *new approach methodologies* (NAMs), including mathematical and computational models, to help study human diseases like AD (AD-NAMs). This narrative review is an approachable starting point for interdisciplinary teams of nutritional scientists, neuroscientists, mathematicians and computer scientists with an interest in developing mathematical or simulation-based AD-NAMs that aim to link diet to AD biomarker pathology.

We introduce the interdisciplinary reader to the three essential areas, including their historical context and contemporary advances, required to chart the further development of simulation-based AD-NAMs: the fundamentals and contextual significance of AD protein biomarker pathology; the history and evidence for dietary influence on that pathology; and an introduction to *network mathematical models* to mathematically analyze and computationally simulate the progression of that pathology. Afterwards, we offer views on bridging the gap between the contemporary approach and those

. that may be used to mathematically and computationally investigate: potential mechanistic links between dietary patterns and AD biomarker pathology; and the potential of dietary patterns to help suppress AD prevalence, at least until reliable pharmaceutical options can be developed

## 1 Introduction

It is well known that Alzheimer's disease (AD), the most common cause of dementia, is a major global health challenge whose severity is only expected to increase (1); currently, around 50 million people have dementia, a number that will nearly triple over the next two decades (2, 3). The AD challenge is coupled with an expected shortfall of 86,000 trained physicians by 2036 (4). There is no estimate for when, or even if, reliable pharmaceutical treatments halting or reversing AD progression may arise, positioning modifiable risk factors as a potential way to reduce AD prevalence until reliable treatments can be developed. Modifiable risk factors for AD are non-genetic, mutable attributes or actions associated with an increased risk of developing AD. As of 2024, 14 such risk factors, accounting for up to 45% of dementia risk, have been identified (3), a third of these risk factors are directly or indirectly related to daily dietary choices.

Given that diet relates to a third of modifiable risk factors (3), one could conjecture that dietary intervention may slow AD onset, progression or both. This conjecture leads to open questions: what biological mechanisms may best link dietary patterns to AD progression; and is AD onset or progression predictable from dietary patterns? These are challenging investigatory questions for which traditional investigatory means may prove incredibly costly in human time, financial cost and animal life. It may prove valuable if there were some quantifiable means to narrow the field of possibilities before investing significantly in experimental costs. The World Health Organization pointed out the pressing need for developing innovative health technologies for AD research a decade ago (5). Today, the National Institutes of Health and the Food and Drug Administration are echoing this call by asking researchers to develop human-relevant *new approach methodologies* (NAMs) (6–8) to improve predictive accuracy, reduce research costs, reduce a reliance on animal testing and expand the set of research tools for difficult biological and medical questions.

The nutritional sciences have a history of using mathematical models to explore potential research paths in otherwise complex landscapes. For example, energy balance models (9) have been used to test mechanistic hypotheses and to predict outcomes regarding relationships between dietary constituents and body weight or body composition. If important AD pathology could be encoded mathematically, those models could be extended to include potential mechanisms linking dietary patterns and AD. Simulation-based NAMs exploring what mechanisms may best explain observational data, and to what extent dietary patterns may ultimately predict or influence AD, then become possible. In fact, a new class of computationally-efficient mathematical model, sharing similarities with energy balance models, can already represent some of the key mechanisms governing the evolution of important AD biomarkers. These models may be a good foundation for a new class of AD-NAMs to research potential links between dietary patterns and AD biomarkers using mathematics and computation.

Developing mathematical and computational AD-NAMs to study how dietary patterns may hasten or delay AD will face some significant challenges. First and foremost, this goal intersects nutritional science, neuroscience, computer science and mathematics, making it highly interdisciplinary. Second, the AD literature, including the view on diet, is voluminous and knowing where to start can be daunting. Third, the nutritional literature is similarly vast with research studies considering a full spectrum of dietary patterns, from broad intake to very specific micronutrients, to study mixtures of both clinical and biomarker-related AD pathology, making it difficult to narrow down and find potential evidence for comprehensive patterns. Finally, it can prove difficult to interpret the current mathematics that describe AD biomarkers, leading to trouble solving these equations computationally or extending them to include new mechanisms. The novelty of this narrative review is that it threads together important historical and contemporary results, at the intersections of AD biomarkers, dietary patterns and network mathematical models, to *provide an accessible starting point for charting the interdisciplinary development of simulation-based AD-NAMs to investigating how dietary patterns may help to delay AD*. Toward this end, we introduce the historical context, essential views and primary findings in three areas: the minimal essentials of amyloid-beta (Aβ) and tau protein (τP) pathology in AD (Section 2); the evidence that dietary patterns do indeed relate to this pathology (Section 3); and a specific class of mathematical methods that have recently been introduced to model this pathology (Section 4). We conclude (Section 5) by highlighting steps and challenges to bridge the gap between the current simulation-based AD-NAMs and those that may be used to study how dietary patterns may help to suppress AD prevalence until reliable pharmaceutical options become available.

## 2 A brief primer on some essential AD biomarkers

This section introduces nutritional scientists, computer scientists and mathematicians to a minimal set of essentials for understanding Aβ and τP pathology in AD; it may also be useful for neuroscience research students who are not yet familiar with AD. The focus is on those essentials that will facilitate research into novel, computational AD-NAMs aimed at researching the potential links, both mechanistic and predictive, between dietary patterns and AD biomarkers using mathematics and computation. This section does not present an exhaustive review of Aβ and τP in AD; rather, it provides the required context for the discussion on dietary patterns (Section 3) and network mathematical models of AD (Section 4) while citing several important publications that the reader may find useful for their own research in simulation-based AD-NAMs. The contents of this section are: a short history of Aβ and τP biomarkers in AD and how they came to be related with clinical presentation (Section 2.1); the contemporary framework for classifying AD based on Aβ and τP biomarkers (Section 2.2); and a short overview of some of the key mechanisms governing Aβ and τP biomarker evolution in AD (Section 2.3).

### 2.1 An abridged history of two primary AD biomarkers and clinical AD presentation

AD was famously described by Alois Alzheimer in 1907 when he reported the analysis of Frau Auguste Deter; Frau Deter was a patient at the asylum in Frankfurt Germany whom Dr. Alzheimer had met, through his long professional collaboration with asylum director Emil Sioli, in 1901 (10). Frau Deter's case wonderfully displayed the duality of AD: the clinical presentation of senile dementia; and the striking protein pathology of τP neurofibrillary tangles (NFTs) and Aβ plaques discovered in her postmortem analysis (11). This dual nature threads its way through our thinking about the disease more than a century later: does one have AD when they show certain signs of clinical dementia or do they have AD when they exhibit sufficient proteinopathy? Sixty years after Alzheimer, Sir Martin Roth quantitatively studied the question by considering the statistical relationship between senile plaques (extracellular Aβ aggregates) and cognitive test performance (12, 13). Around that same time, Roth was collaborating with the neuropathologist Sir Bernard Tomlinson to consider an accounting of τP NFT in the brains of patients with and without dementia (14). This collaboration, and a rigorous pursuant study by Gordon Wilcock and Margaret Esiri in the 1980s, demonstrated that τP NFT were highly correlated with dementia while Aβ plaque correlation was weaker (15).

Early statistical studies correlating Aβ and τP with clinical symptoms may have strongly influenced the view of AD as a combined clinical-pathologic entity (16, 17). The official diagnostic criteria for AD originated 1984 and cited the early work of Roth (18). A diagnosis of AD is based primarily on cognitive, behavioral and functional impairment measured by a combination of noted shifts in lifelong patterns of memory and executive function, or performance on neuropsychological exams relative to expected outcomes. Once preliminary criteria are met, probable AD is diagnosed based on genetic evidence or on the decline in memory and learning and whether cognitive decline is steady. If the bar for probable AD is not met, then possible AD is the diagnosis (19); an AD diagnosis is confirmed by post mortem autopsy. These criteria were designed based on the prevailing view that AD neuropathology, like Aβ and τP aggregates, were in tight, virtually synonymous correspondence with clinical symptoms; patients without AD dementia were anticipated to be free of AD pathology while patients who had AD dementia were thought to also have developed AD pathology (16).

The assumption of tight, or synonymous, correspondence between clinical symptoms and AD neuropathology is not sound. It is now well known that diffuse Aβ plaques occur in cognitively asymptomatic patients in an extended preclinical phase (17, 20). About 30 years after the NINCDS-ADRDA criteria, the role of AD protein pathology in slowly advancing the disease from a pre-clinical to a clinical stage was receiving attention (16); as any AD intervention would need to identify biologically defined targets, the push to see AD in terms of biomarkers and to classify clinical stage separately was already underway (17).

### 2.2 The A/T/N framework for AD diagnosis and staging

The “*amyloid cascade hypothesis*” of AD (21–23) famously postulated that “*the deposition of A*β *protein is the causative agent of Alzheimer's pathology and that NFTs, cell loss, vascular damage and dementia follow as a direct result of this deposition*.” Whether Aβ or τP misfolding and aggregation are the etiological factors of AD is now debated; what is not debated is that Aβ and τP pathology are central biomarkers of AD and AD progression. The A/T/N framework advances a view of AD based on the primary biomarkers of Aβ and τP pathology; the framework describes diagnostic criteria in addition to criteria for disease staging with a separate classification that tracks clinical progression. The A/T/N criteria are the culmination of over a decade of analysis, consideration and planning (16, 17, 24, 25) and offer an invaluable tool for AD research.

The A/T/N emphasizes measurable AD biomarkers. AD biomarkers are separated into 4 categories: category “A” is related to Aβ pathology; category “T” is related to τP pathology; category “N” is related to pathology related to neurodegeneration; the final category does not have a letter label, but includes non-specific processes, like neuroinflammation, and biomarkers of non-AD copathology like vascular brain injury and levels of α-synuclein. The A and T categories make up the so-called *core biomarkers* and are the most important for AD diagnosis and staging. Core biomarkers are further categorized as either *core 1* or *core 2* with core 1 establishing the foundation for diagnosis and core 2 establishing additional means to assess progression. Biomarkers in the “N” category, or the unlabeled category, are reportedly inconsistent across patients but do provide prognostic value; thus, they add context to the core assessments (25). Most importantly, the A/T/N diagnosis of AD requires abnormal Aβ, established through either positron emission tomography (PET) imaging or through abnormal levels of τP and Aβ detected in a cerebrospinal fluid (CSF) sample [(25), Table 2]; both of these measures are mildly invasive but the technology to detect Aβ and τP in plasma with very high accuracy is making its way to points of care.

**Figure 1:**
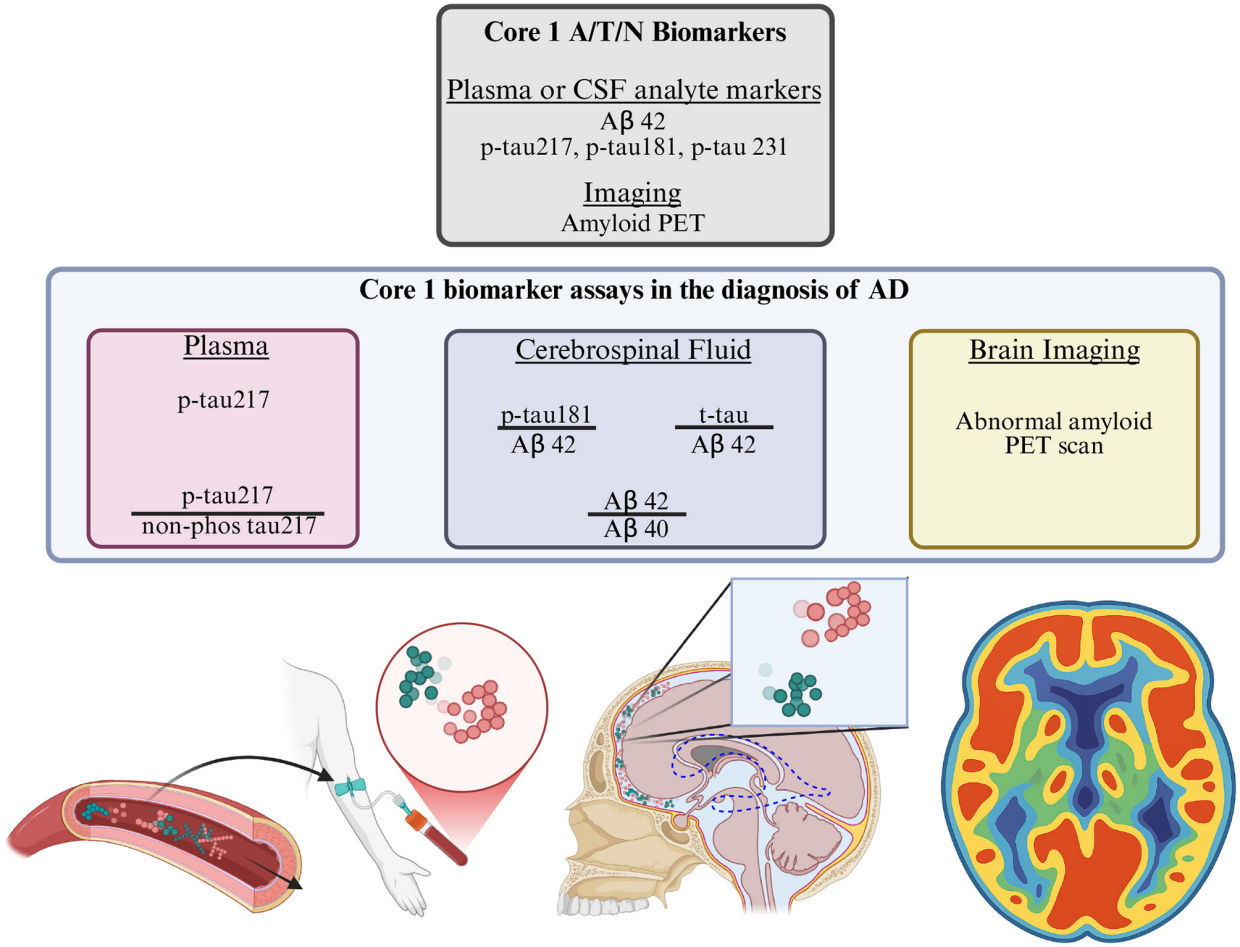
Core 1 biomarkers are defined by Aβ (red spheres) and τP (green spheres), from plasma, CSF and imaging. Core 1 biomarkers are central to AD diagnosis in the A/T/N framework and consist of: tau phosphorylated at site 217 (ptau217) in plasma **(bottom left)**; the 40 and 42 amino acid Aβ peptides (Aβ40, Aβ42), tau phosphorylated at site 181 (p-tau181) and total tau (t-tau) content in CSF **(bottom middle)**; and brain Aβ aggregate levels measured by Aβ PET imaging **(bottom right)** [(25), Tables 1, 2].

The A/T/N establishes both AD biomarker and clinical staging. The original 1984 view of the NINCDS-ADRDA criteria, that clinical dementia is synonymous with biomarker pathology, is no longer accepted. In particular, abnormal Aβ pathology can exist in clinically asymptomatic cases; conversely, coexisting conditions can shift clinical symptoms earlier or later in the disease process whereas A and T biomarkers display prototypical trends [(25), Figure 1]. Core 1 biomarkers (Figure 1), i.e., plasma and CSF levels of particular Aβ and τP analytes or Aβ PET, currently provide the earliest means of AD detection [(25), Table 1]. After AD is detected, core 1 and core 2 biomarkers, together, give an idea of what stage the patient is in based on their level of τP pathology via imaging or fluid analytes [(25), Tables 3–5]. Finally, the A/T/N also proposes seven different clinical stages which, when coupled to biomarker staging, yields a total of 17 possible AD states and a step-wise trajectory expected to apply to most patients [(25), Tables 6, 7]. In summary, the A/T/N disentangles the clinical AD perspective, fraught with comorbidities and patient-specific variation, from the underlying measurable neuropathology of AD biomarker status. In doing so, it defines actionable intervention targets for AD, a clear AD diagnostic criteria and comprehensive staging categorization.

### 2.3 A brief overview of Aβ and τP proteinopathy in AD

The core 1 and core 2 biomarkers of the A/T/N framework are all measures of Aβ and τP pathology. At a high level, the Aβ and τP proteinopathy in AD can be conceptualized as governed by a tripartite paradigm: the production of Aβ and τP species that can aggregate; brain clearance mechanisms that manage Aβ and τP levels; and the prion-like reproduction and spreading of aggregated pathology.

#### 2.3.1 The production of aggregation-competent Aβ and τP

In 1987 it was discovered that Aβ is derived from APP, an evolutionarily conserved transmembrane protein with physiological purposes including cell growth, maturation, proliferation, survival and repair, among others (26, 27). Aβ pathology ultimately arises from APP processing by secretases. APP processing has been covered at great length (26–37). Briefly, different secretase types cleave APP protein in different places; the sequential ordering in which secretase types cleave APP is called an APP processing pathway. The “amyloidogenic pathway,” β secretase followed by γ secretase, produces Aβ peptides that can aggregate together to form the infamous plaques associated with AD dementia; the “non-amyloidogenic” pathway, α followed by γ secretase, does not produce the aggregation-prone Aβ peptide. The two most common amyloidogenic forms of Aβ are Aβ40 and Aβ42. Aβ42 accounts for around 20% of the overall Aβ production, is more prone to aggregation and its aggregates are more neurotoxic (28).

The NFTs first noticed by Alzheimer (11) were aggregates of the τP protein. τP, like Aβ, is a critical brain protein that helps give structure to cells, has been implicated in the brain's response to insulin, cell-cycle maintenance, neurogenesis and synaptic function among others (38). Unlike Aβ, τP is not a downstream cleavage product of another protein. τP arises from the alternative splicing of the MAPT gene, creating up to six different (mRNA) blueprints used to build the final τP protein. τP plays an important role in regulating microtubules in cells (38–40) and is constantly being modified in order to carry out this function. Some modifications, when they occur in the right order, promote τP to detach from microtubules and aggregate, eventually forming the NFT hallmarks of AD (41–44). A recent post-mortem analysis of 19 patients suggested a staging of τP PTM relevant to AD: (A) initial phosphorylation; (B) enhanced phosphorylation; (C) initial acetylation and ubiquitination; and (D) enhanced acetylation and ubiquitination [(44), Figure 5]. As τP moves down this chain of modifications, it becomes more likely to aggregate and form fibrils and NFT.

Plaques of Aβ and NFTs of τP are the canonical hallmarks of AD protein pathology. Broadly speaking, aggregation-capable Aβ or τP monomers can form oligomers (45–47); oligomers consist of a small number of monomers and are named according to their monomer count, such as a dimer (2 monomers) or a trimer (3 monomers), etc. Some oligomers can spur further aggregation to protofibrils which, in turn, can form fibrils that aggregate to form the plaques and NFTs of AD (47). Growing evidence supports the hypothesis that oligomers and protofibrils are the most cytotoxic aggregated Aβ or τP species, that Aβ plaques and τP NFT are likely nontoxic, while monomers and fibrils are only mildly toxic or non-toxic (48–52). Some studies suggest that oligomeric Aβ may have a hormetic effect, being beneficial at lower concentrations but detrimental at higher ones, suggesting that concentration may mediate at least some oligomeric species' cytotoxicity and that low concentrations of those Aβ oligomers are unlikely to be cytotoxic (53–55); τP oligomers may exert toxicity in a dose-dependent manner so that low concentrations, even if not beneficial, may not be harmful (56).

#### 2.3.2 Brain clearance manages Aβ and τP

Aβ, τP and their aggregates, especially small oligomers, are constantly produced in the brain. While early onset AD is thought to originate from overproduction, sporadic AD, the most common form of AD, may originate from deficits in brain clearance (57–59). Mechanisms of Aβ and τP brain clearance have been discussed in many reviews (33, 58, 60–65); we will briefly overview the essentials. There are two primary clearance mechanisms: intracellular or extracellular hydrolytic degradation; and transport out of the brain followed by proteolysis in peripheral organs (58, 63, 64). Intracellular Aβ or τP degradation can occur via the proteasome, with or without ubiquitin mediation, the endosome-lysosome or autophagy-lysosome pathways or via cytosolic proteases like insulin-degrading enzyme (Aβ only) (58, 61, 63, 65).

Extracellular Aβ and τP are also cleared; they can, of course, reengage the intracellular space, of neurons or glia, via autophagy, endocytosis or chaperones and be processed by intracellular mechanisms. Beyond this, extracellular proteases like neprilysin, insulin degrading enzyme and matrix metalloproteases can degrade Aβ in the extracellular space (33, 63, 64). Extracellular Aβ can exit the brain into the bloodstream by way of transporters like those in the ATP binding cassette or low density lipoprotein receptor families; Aβ can also be brought back into the brain, from the blood, by receptors for advanced glycosylation end-products (33, 63, 64). Finally, the glymphatic system also participates in Aβ and τP clearance. Briefly, CSF enters the brain along the perivascular spaces of cerebral arteries, is transported into the parenchyma where it picks up Aβ and τP before exiting along perivenous spaces, delivering its contents to the peripheral lymphatic system (60, 62).

#### 2.3.3 The prion-like hypothesis of AD

The prion-like hypothesis is a fundamental tenant of AD research and implies that Aβ and τP do more than simply aggregate in place in AD; in Section 4, we will see that the prion-like hypothesis strongly motivates the use of *network mathematical models* to study AD biomarker evolution. The prion-like hypothesis has its roots in the discovery of prions. In the mid 1980s PrP^*Sc*^, a misfolded form of the PrP protein, was found to be both the progenitor and propagator of scrapie in sheep; cytotoxic, misfolded PrP^*Sc*^ templated its own replication, converting physiological PrP, spread to connected regions and aggregated (66–70). The relationship between Aβ aggregates in AD and PrP^*Sc*^ aggregates in the neurodegenerative Kuru and Crutzfeldt-Jakob diseases led to the postulate that physiological Aβ may also act in a prion-like manner, possibly leading to AD (69). The hypothesis that Aβ may behave like PrP^*Sc*^ implied: that some Aβ structures (oligomers, fibrils, plaques, etc) should be integral to AD progression; that these structures should be a template for self-reproduction from non-AD associated Aβ; and that this action should propagate AD pathology, in particular to connected regions.

Evidence for the prion-like hypothesis in AD, that Aβ and τP could spread from one brain region to another, potentially along axonal projections, and reproduce through autocatalytic templated misfolding, began mounting in the early 1990s. Steven Arnold's histopathological analysis of τP NFT distribution suggested a predictable regional NFT pattern; Arnold drew repeated attention to the cortico-cortical axonal connectivity of τP affected regions (71). Shortly afterwards, Heiko and Eva Braak conducted another histopathological analysis of τP and Aβ pathology in 83 AD brains, finding evidence for a 3-stage progression of Aβ pathology [(72), Figures 1, 4] and a 6-stage progression of τP pathology [(72), Figures 1, 4]; pathological aggregation was amplified when moving from one stage to the next [(72), Figures 5–10]. There is now a good deal of evidence that both Aβ and τP behave in a prion-like manner in AD (73–79). Broadly speaking: Aβ or τP seeds, i.e., misfolded aggregates of a sufficient size to act as a template, become sites for creating new seeds from an existing population of Aβ or τP monomers; these Aβ and τP seeds spread to axonally connected regions, create more misfolded seeds there, which continue to spread and further AD neurodegeneration.

## 3 Dietary patterns may influence AD risk and AD biomarkers

The literature on diet and its potential relationship to AD is extensive. The interdisciplinary research team interested in developing novel AD-NAMs, to assist in diet-related AD research, may find it daunting to approach the problem due to a lack of clear starting points. For example, an AD-NAM designed to predict incident AD risk in a large population adhering to broad categorical dietary patterns, like the Western or Mediterranean diets, may need to consider different approaches than an AD-NAM that would be used to study micronutrient effects on measured Aβ levels in single patients. Though it remains an open question as to what, specifically, these differences may be, a reasonable interdisciplinary team would likely begin by searching for evidence at these respective macro and micro dietary pattern levels. This section introduces neuroscientists, computer scientists and mathematicians to a set of approachable starting points that we believe are important for novel AD-NAM research. This section may also be useful for nutritional science research students who are not yet familiar with AD.

**Figure 2:**
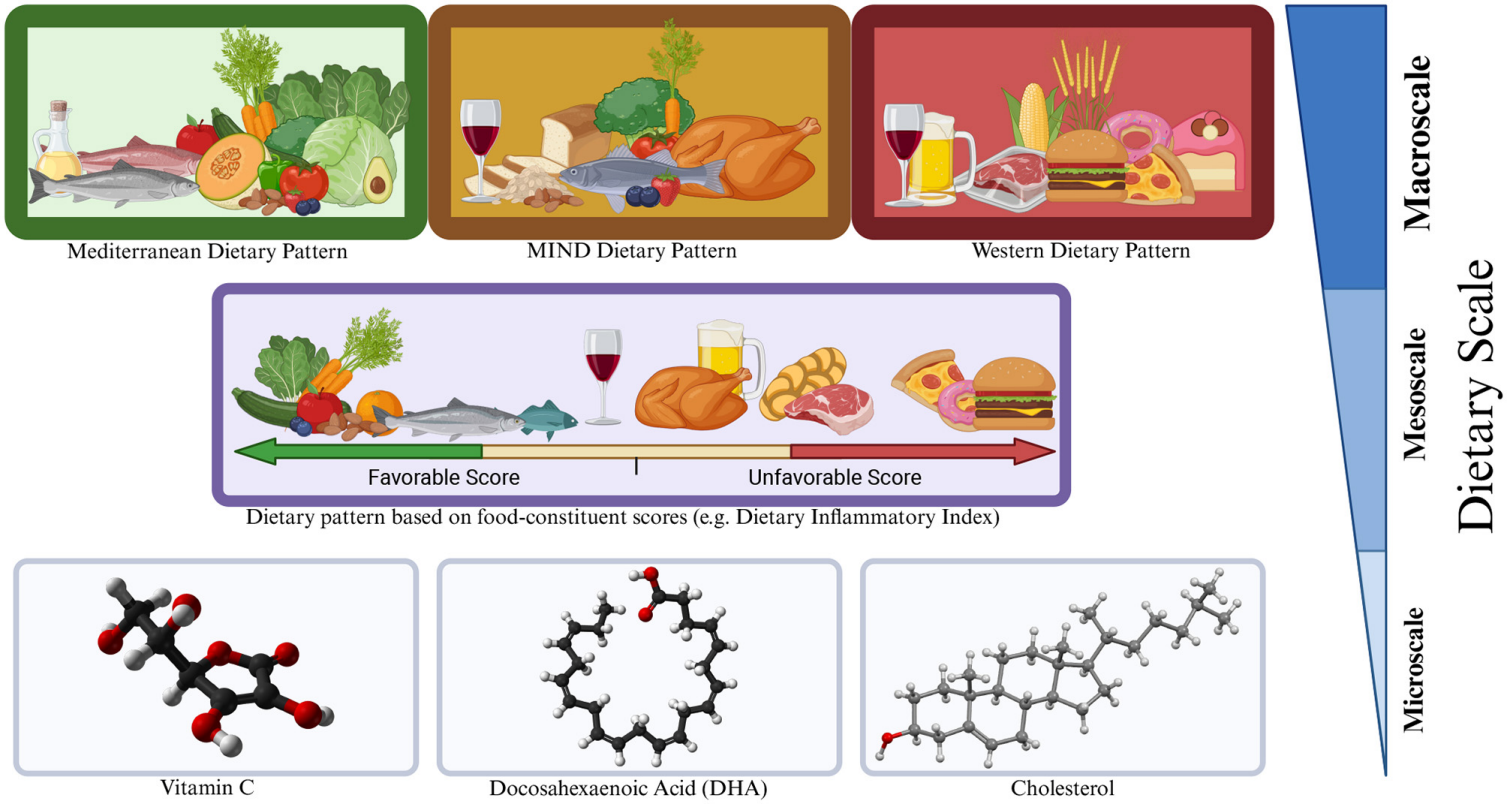
A three-scale conceptual framework for discussing the influence of dietary patterns on AD. Macroscale patterns **(top)** are defined by broad categorical intake; mesoscale patterns **(middle)** are defined by constituent scores (kcal, fiber, fat, vitamin C, etc.) aggregated to quantify a total intake; microscale patterns **(bottom)** track the intake of single constituents (vitamin C, DHA, etc.).

To simplify our presentation, this section uses our own *descriptive convention* to categorize dietary patterns into 3 classes that reflect their underlying process of assessment and their potentially different research motivations for developing computational AD-NAMs. Reusing terminology from the meteorological and atmospheric sciences, we refer to these classes as the dietary macroscale, mesoscale, and microscale (Figure 2). The *dietary macroscale* looks to how adherence to a pre-defined class of foods, generally measured by consistently meeting a categorical set of portion allotment each day or week, impacts an outcome of interest without being overly specific regarding the constituents within those suggested categories (Section 3.1). The *dietary mesoscale* quantifies the effect of an intake pattern on an outcome of interest by examining the balance of a predetermined list of constituents but is otherwise agnostic about the intake pattern (Section 3.2). Finally, the *dietary microscale* concerns itself with measuring the effects of a specific constituent, such as vitamin A or eicosapentaenoic acid, on an outcome of interest (Section 3.3).

At each dietary scale, we briefly summarize key historical elements before discussing the results for two questions that we believe are central to efforts to develop AD-NAMs: which, if any, dietary patterns show evidence of modifying incident AD risk; and which, if any, dietary patterns show evidence of influencing AD biomarkers of the A/T/N framework (Section 2.2). Due to the nature of human studies, the literature on these two questions has demonstrated conflicting results. These conflicts have now mostly been reviewed, at length, many times. Thus, we do not endeavor yet another exhaustive review of the many individual studies on broad or specific dietary constituents that may relate to AD. Instead, at every possibility, we present the reader with the results of recent pooled meta-analyses, and comprehensive umbrella reviews, on these topics. To keep the discussion succinct, we state those findings for which significant evidence was achieved from pooled results in addition to the number of studies and the measure of heterogeneity, whenever available. In this way, effective starting points, based on a sense of statistical consensus, are advanced and the interdisciplinary team interested in study specifics can consult the cited meta-studies. In this way, we present interdisciplinary teams with essential, approachable starting points for efforts aimed at developing AD-NAMs, especially those based on mathematical models, to investigate the links between dietary patterns and AD.

### 3.1 The dietary macroscale and AD

This section discusses how the *dietary macroscale* may affect AD risk and AD biomarkers as described by the A/T/N framework (Section 2.2). Dietary patterns at the dietary macroscale are typically assessed by measuring adherence to pre-defined classes of foods, like “baked goods” or “seafood,” through counting servings per day, week or month. Common examples include the Western Diet (WD), Mediterranean Diet, DASH Diet, and the MIND Diet. The WD, in particular, attracted early attention with Newman's hypothesis that it may actually cause AD (80). Soon after, follow-up studies began to connect a western diet (WD) to AD risk (81–83). The WD is high in processed foods, sugars, salt, trans, and saturated fats and low in polyunsaturated fats, vegetables, and fruits (84–88). The WD is linked to inflammation and oxidative stress and, in excess, can lead to obesity, high levels of circulating insulin, insulin resistance and type 2 diabetes mellitus (87, 89). Oxidative stress, inflammation, and insulin dysregulation are mechanistic factors linking diet to chronic neuronal stress and to AD (61, 90).

AD is considered to be one of the “diseases of civilization” that arose concomitantly with a western lifestyle and the macroscale WD pattern of eating. However, the understanding that the WD may act to increase incident AD risk took decades to develop. It began in 1995 with an age-standardized comparison of AD prevalence between elderly residents of Ibadan (Nigeria) and Indianapolis of similar ethnic origin; AD prevalence in the Indianapolis population was 4.43 times higher (82). In 1996, another age-standardized study compared elderly Hisayama (Japan) men to their ethnic counterparts in O'ahi (Hawaii); AD prevalence was 3.6 times higher in O'ahu (83). These studies prompted researchers to suspect “environmental or cultural exposures” associated with migration to the United States. Motivated by these studies, William Grant published the first link between diet and AD risk in 1997. Grant found that total caloric intake and total dietary fat, elevated in WD populations, were significant in increasing AD prevalence while cereals and fish reduced AD risk (81). Two years later, Grant added: that it was not clear if whole grains and cereals were protective or may be displacing foods that increased AD risk; that some fats, like omega-3 in fish, seemed to reduce AD risk while others, like arachidonic acid or an imbalanced omega-3/omega-6 ratio, may increase AD risk (91). He also pointed toward the possibility that dietary-induced inflammation and oxidative stress may act to enhance the risk of AD.

Grant's early work helped to point a path forward and increasing evidence suggests that that some macroscale dietary patterns may indeed reduce AD risk. These include the Mediterranean diet (MED) (92); the Dietary Approaches to Stop Hypertension (DASH) diet (93); and the Mediterranean-DASH Intervention for Neurodegenerative Delay (MIND) (94) diet. A relationship between high adherence to MIND, MED, or DASH and a reduction in AD risk continues to appear in cross-sectional and longitudinal studies. A recent systematic review compiled an extensive list of studies that considered the influence of MED, MIND, and DASH on a spectrum of cognitive impairments, including AD risk (95). A case-controlled MED cohort study found evidence for a 19% reduction in AD for each increment of MED score while high MED adherence was reported to reduce AD risk by at least 60% compared to low MED adherence. [(96), Table 3]. Several longitudinal studies offered evidence for a possible reduction in AD risk for high adherence to MED, MIND, and DASH. Five longitudinal studies using the MED dietary pattern reported reductions in AD risk ranging from 9% to 54% depending on whether a continuous (per increment) MED score or a tertile comparison was used (97–101). A longitudinal study reported that high DASH adherence may reduce AD risk by 39% and the MIND diet may reduce AD risk by 35% to 53% depending on the adherence tier position in a tertiary analysis (98). A recent pooled meta-analysis of 11 cohorts from 3 studies also concluded that high MIND adherence reduced (11 studies, *I*^2^ = 35%), incident dementia, including AD dementia, risk by 17% compared to low (WD-like) adherence [(102), Figure 2]. It is worth noting that results can vary regionally. For instance, MED adherence was not associated with AD dementia in a number of longitudinal French, Swedish and Australian studies (95), possibly reflecting that diet is related to a third (2, 3), but not all, of the modifiable AD risk factors which may themselves vary regionally or that their regional dietary patterns may already confer sufficient reductions in AD risk. Conversely, studies and meta-analyses using cohort data from the UK and USA (98, 102) did find evidence for changes in AD risk, suggesting that diet may be more prominent among modifiable AD risk factors in those locations or that these regional diets may possibly be associated with an increase AD risk.

Macroscale dietary patterns may also be associated with AD biomarkers. A recent umbrella review amalgamated the reports of meta-analyses and systematic reviews that considered the mediating effects of diet on biomarkers of cognitive decline, including AD (103). The reported results suggested that significant associations may exist between: reduced hippocampal volume and WD adherence; reduced thickness of the frontal cortex and WD adherence; increased hippocampal volume and MED adherence; increased Aβ deposition with (high glycemic) WD adherence; reduced Aβ deposition with MED adherence (for Pittsburgh Compound B imaging radiotracer studies but not for studies using other Aβ radiotracers, 4 studies); higher baseline brain metabolism and MED adherence; and a decreased rate of brain metabolic decline and MED adherence.

The gold standard of AD biomarker assessment is postmortem analysis, though these studies are less encountered in the nutritional literature. The Rush Memory and Aging Project tracked both dietary intake and a postmortem analysis of several participants, leading to four recent publications. MIND dietary score was correlated with better cognitive function before death and slower cognitive decline, and these associations remained significant after controlling for postmortem confirmation of AD (104, 105). MIND and MED, diet scores were significantly and inversely related to postmortem total brain Aβ levels and global levels of combined (Aβ and τP) AD pathology, but not τP NFT pathology alone (106). A postmortem RNA-seq analysis also suggested that MIND dietary score may be associated to a set of 50 genes; the strongest associations were related to gene expressions mediated by (positive association) educational attainment and (negative association) AD-related white matter structural changes (107). Taken together, there may indeed be good evidence to suggest that macroscale dietary patterns could be related to AD biomarkers of the A/T/N framework. It is not fully clear, from these studies, what mechanisms, like oxidative stress or inflammation, may yet link the dietary patterns to AD biomarker outcomes.

### 3.2 The dietary mesoscale and AD

This section discusses how the *dietary mesoscale* may affect AD risk and AD biomarkers as described by the A/T/N framework (Section 2.2). Dietary patterns at the dietary mesoscale are, in a sense, an attempt to instrument a macroscale pattern for research use. Mesoscale dietary patterns assign an overall score to an intake by summing over a set of constituents. For a diet concerned with oxidative stress, we may assign a score, *N*, to “nut consumption in g/day” that relates nut consumption to markers of oxidative stress measured in the blood (108, 109). Consuming 200g of nuts in one month would add *N*(200/30) to that “oxidative stress” dietary pattern. Constituents at the mesoscale can be broad, like “nuts” or narrow, like “almonds” or even “vitamin D3.” This differs from macroscale diets, like MED, which assign pre-defined adherence values, like 0, 0.5, or 1, to broad categories, like “consumed 5 daily servings of fruits and vegetables,” based on how closely that goal was attained (94, 110).

Studies based on the dietary inflammatory index (DII) demonstrate that the dietary mesoscale could impact AD risk. The DII originated as a means to study diet-mediated inflammation (108, 109). It was revised (111) to the energy-adjusted DII (E-DII) to follow existing practices for assessing energy intake in epidemiological studies (112, 113). E-DII values for individual dietary constituents are not directly available in the literature due to a patent by the original authors; however, unadjusted DII values are available alongside a helpful description of score construction (109). Many studies report that the following nutrient density was used to determine their E-DII scores:


$$\text{Nutrient per 1000 kCal}=\frac{\text{Total Nutrient}}{\left(\frac{\text{Total kCal}}{1000}\right)}=\frac{\text{Total Nutrient}}{\text{Total kCal}}\times 1000.$$

**Figure 3:**
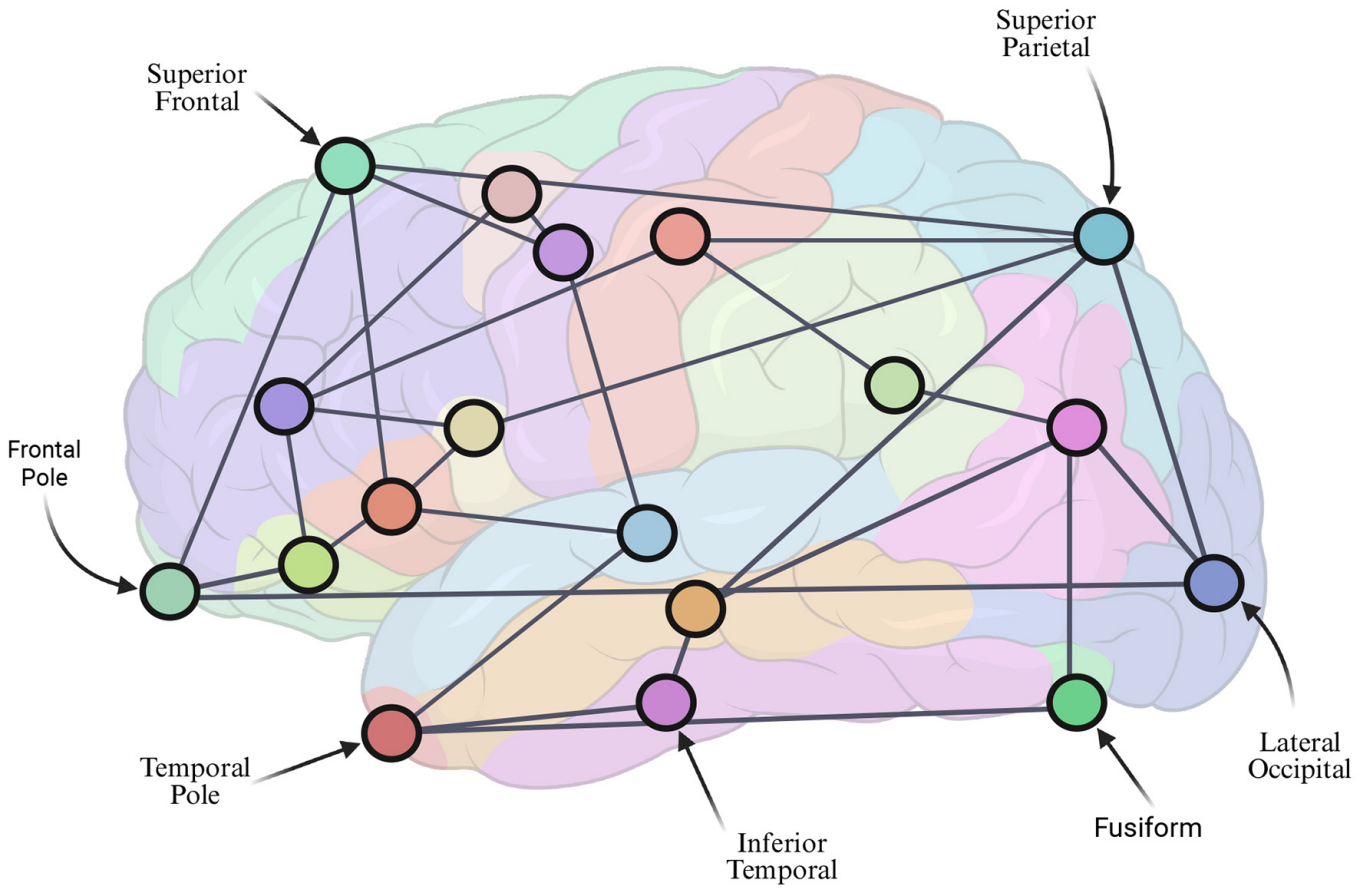
A stylized illustration of a brain structural connectome graph. A brain T1 MRI sequence is segmented into anatomical regions (colored regions in background, some anatomical labels are shown for emphasis) to define the vertices (circles in foreground) of the brain connectome graph. The vertices are connected by edges (gray lines) that represent the axonal connectivity estimated by a tractography method applied to a diffusion-weighted MRI.

The first DII study to consider the AD pathway analyzed a cohort of American women; it established that the incidence of mild cognitive impairment (MCI) or probable dementia was 27% higher in patients with high DII scores, compared to those with low scores, with fewer years without impairment [(114), Table 4, Figure 2]. More recently, two additional large UK cohort studies (UK Biobank) found interesting relationships between DII and AD risk. One found that that high (Q5) DII subjects were 66% more likely to develop AD compared to mid (Q3) DII subjects, controlling for age and sex, whereas high (Q5) DII subjects were 59% more likely to develop AD compared to low (Q1) DII subjects when controlling for age, sex and 17 other covariates (115). This work also found that DII had subgroup effects, on AD risk, related to both sex and education [(115), Figure 3] and that the relationship between DII and *all cause dementia* was nonlinear. A second large UK Biobank study also found a significant nonlinear relationship, but this time between DII and AD risk [(116), Figure 2]. In particular, above a threshold DII score (1.3), each unit of DII increased incident AD risk by 39% [(116), Table 3]. However, below this threshold the DII was not significantly associated with AD risk and was not overall significantly associated with AD risk. This result suggests that the relationship between dietary-related inflammation and AD risk may be nuanced and resilient to some initial level of dietary-related inflammation but is significantly, and nonlinearly, increased once dietary-related inflammation is too high. This group also found subgroup effects mediated by BMI and education but not by sex, partially conflicting the previous group's findings.

The previous studies were carried our in the USA and the UK but relationships between the mesoscale DII and incident risk along the AD pathway appear to be consistent in both Chinese and Greek cohorts.

Two Chinese population studies found that high scores were, respectively, 1.46 (E-DII) and 1.23 (DII) times more likely to develop MCI (117, 118); a similar Chinese population study showed that high DII conferred a 50% increased risk of incident MCI (119). Five years later, the mediating effect of DII was studied in a Mediterranean cohort (120); participants in the highest tertile of DII score were about 3 times more likely to develop dementia, the strong majority of which were AD cases, than those with the lowest DII score (121). Finally, a recent meta-analysis of inflammatory diet and cognitivie function found a 33% increase to incident MCI risk (4 studies, *I*^2^ = 0%, *p* = 0.39) and a 34% increase in incident MCI plus dementia risk (5 studies, *I*^2^ = 36%, *p* = 0.18) with a pro-inflammatory DII or E-DII score [(122), Figure 2].

Evidence for mesoscale dietary patterns and AD biomarkers is limited. A large UK biobank study found evidence of an association between increasing increments of DII score and a decrease in gray matter hippocampal volume [(116), Table 4] after adjusting for 13 covariates. Aside from this, we found only one other related, albeit indirectly, study. E-DII score was significantly correlated with 55 immune proteome constituents in blood samples. The study identified six of these proteins (CXCL10, CCL3, HGF, OPG, CDCP1, and NFATC3) as significantly associated with increased odds of cognitive impairment using data from external cohorts (123). Moreover, Aβ42/40 levels were significantly correlated with CXCL10, CCL3, NFATC3, HGF, and OPG while NfL was significantly correlated with CXCL10, CCL3, CDCP1, and OPG; brain atrophy was significantly correlated with OPG, CCL3, and CDCP1. However, statistical significance is not transitive and the direct effects of DII on AD biomarkers remains an open question.

### 3.3 The dietary microscale and AD

Around the time that Grant linked diet to AD, vascular factors, and oxidative stress were postulated as contributors to AD pathogenesis. These two views led researchers to ask whether specific micronutrients may mitigate AD risk, including: B vitamins, for their relation to elevated plasma homocysteine in vascular disease; and Vitamins C and E, which are potent antioxidants. Martha Morris and Robert Clarke offered some of the first results. Morris' cohort study showed no cases of AD incidence in a subgroup supplementing with Vitamin C or Vitamin E despite the expected rate being between 9% and 14% but the cohort was too small for significance (124). Clarke showed that low serum folate (B_9_) conferred a significant 2.3 fold risk for clinically diagnosed AD while low B_9_ or B_12_ significantly increased the risk for histologically confirmed AD by 3.3 or 4.3 fold, respectively [(125), Table 2].

The early work of Morris and Clarke opened the door to research into the role of micro- and macronutrients, as opposed to whole diets, in AD. About a decade later, Morris published a review of several cohort studies that included a view on dietary fats and pointed out conflicting AD-risk results for vitamin E and vitamin C (126). Given the sheer abundance of micro- and macronutrients, these early perspectives paved a suggestive path forward. A few years later, a systematic review of AD patient studies concluded that plasma levels of vitamins A (9 studies, *I*^2^ = 87%), C (8 studies, *I*^2^ = 88%), E (20 studies, *I*^2^ = 87%), B_12_ (37 studies, *I*^2^ = 87%), and folate (31 studies, *I*^2^ = 88%) were significantly lower in patients with AD but vitamin D (5 studies, *I*^2^ = 95%) did not achieve significance (127). A recent meta-analysis of studies of vitamin deficiencies, between AD and control patients, may shed further light on this early work (128). Vitamins A (9 studies, *I*^2^ = 2.4%), C (8 studies, *I*^2^ = 90.7%), E (21 studies, *I*^2^ = 90.2%), and folate (31 studies, *I*^2^ = 93.3%) all showed significant reductions in AD patients versus controls; the reported study heterogeneity was substantial in all analyses except for Vitamin A. An additional network meta-analysis (*I*^2^ = 91.9%) suggested that B_12_ may also be reduced in AD patients. Overall, the pooled results of the studies showed that deficiencies were, in order from greatest to least, vitamin C, then D, folate, E, A, and *B*_12_. Some limitations to these analyses were also reported. In addition to the substantial inter-study heterogeneity, mentioned above, for most of the vitamins, a meta-regression also found that age may account for the vitamins C and E deficiencies, but not the others, in the AD group while a publication bias analysis found that biases in the vitamin E and folate publications [(128), Sections 3.3–3.6].

**Figure 4:**
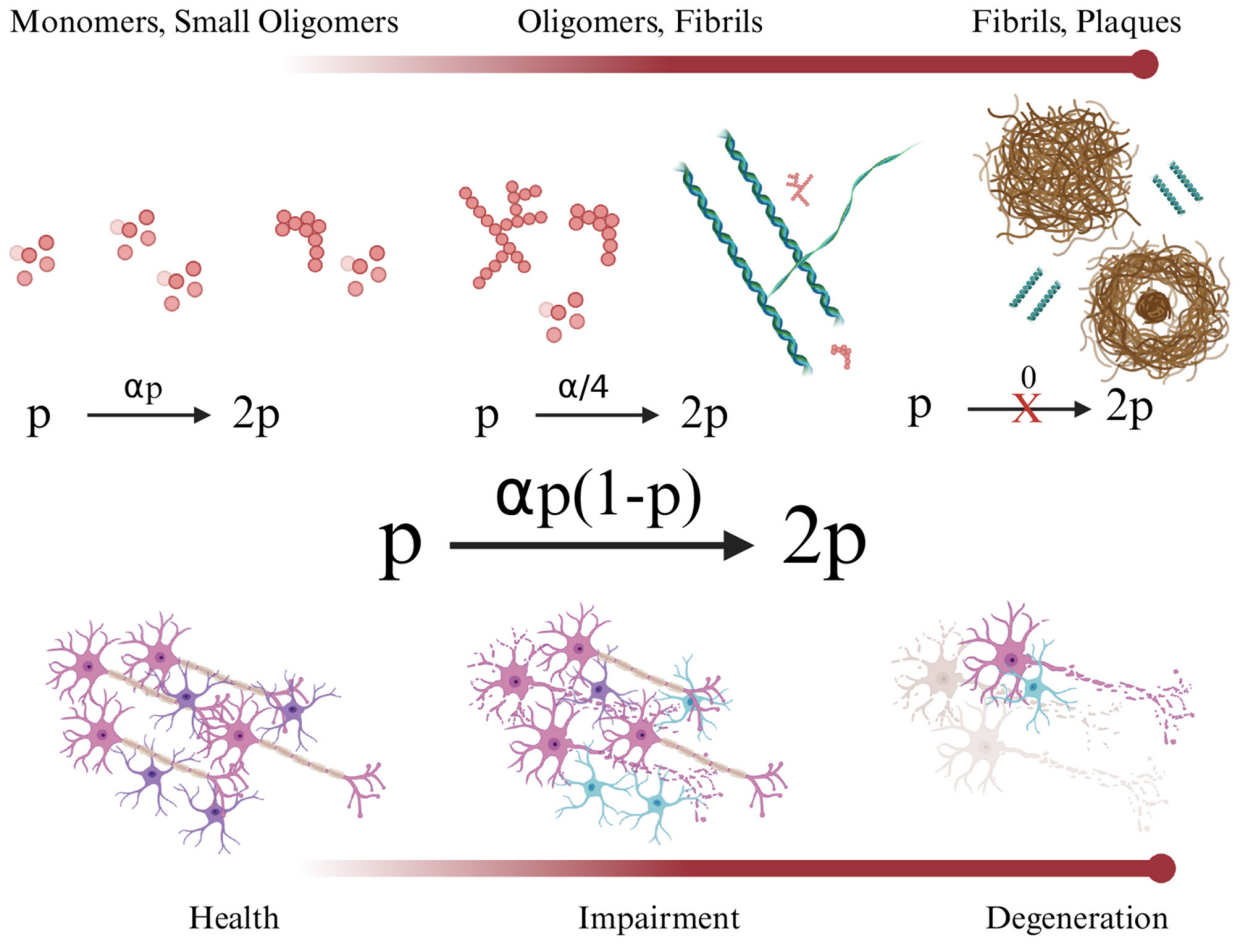
The Fisher-Kolmogorov model states that Aβ (or τP) pathology (*p*) expands exponentially when small oligomers dominate (**left**, 0 < *p*≪1). As aggregates damage cells and fibrils form, new aggregates are made at a linear rate (**middle**, *p*≈1/2). As neurons die en masse, Aβ production is significantly attenuated; thus, further misfolding and aggregation effectively halt (**right**, *p*≈1).

Nutrient deficiencies amongst patients with AD motivated incident AD risk research at the microscale; these results can be nuanced. For instance, it may be unclear whether vitamin A, or its precursors, reduces incident AD risk. A recent systematic review and meta-analysis concluded that low serum levels of α-carotene, β-carotene, and β-cryptoxanthin, all retinol precursors, were not associated with AD status (129) while the non-vitamin A carotenoids lutein and zexanthin were. Two additional meta-analyses found no evidence (6 studies, *I*^2^ = 0%) that dietary or supplemental vitamin A [(130), Figure 4, Table 2] or (5 studies, *I*^2^ = 25.2%) β-carotene [(131), Figure 2] reduced incident AD risk. However, vitamins E and C both showed separate associations with reduced incident AD risk in both meta-analyses; vitamin E was associated with 23%–24% risk reduction (12 studies, *I*^2^ = 20.9% and 12 studies, *I*^2^ = 54%) while vitamin C was associated with a risk reduction of 19% (11 studies, *I*^2^ = 0% and 11 studies, *I*^2^ = 37.9%) (130, 131). These studies did not find an association between combining vitamins E and C and reduced AD risk. Several large, recent meta-analyses have considered the effect of B vitamins on AD risk.

**Figure 5:**
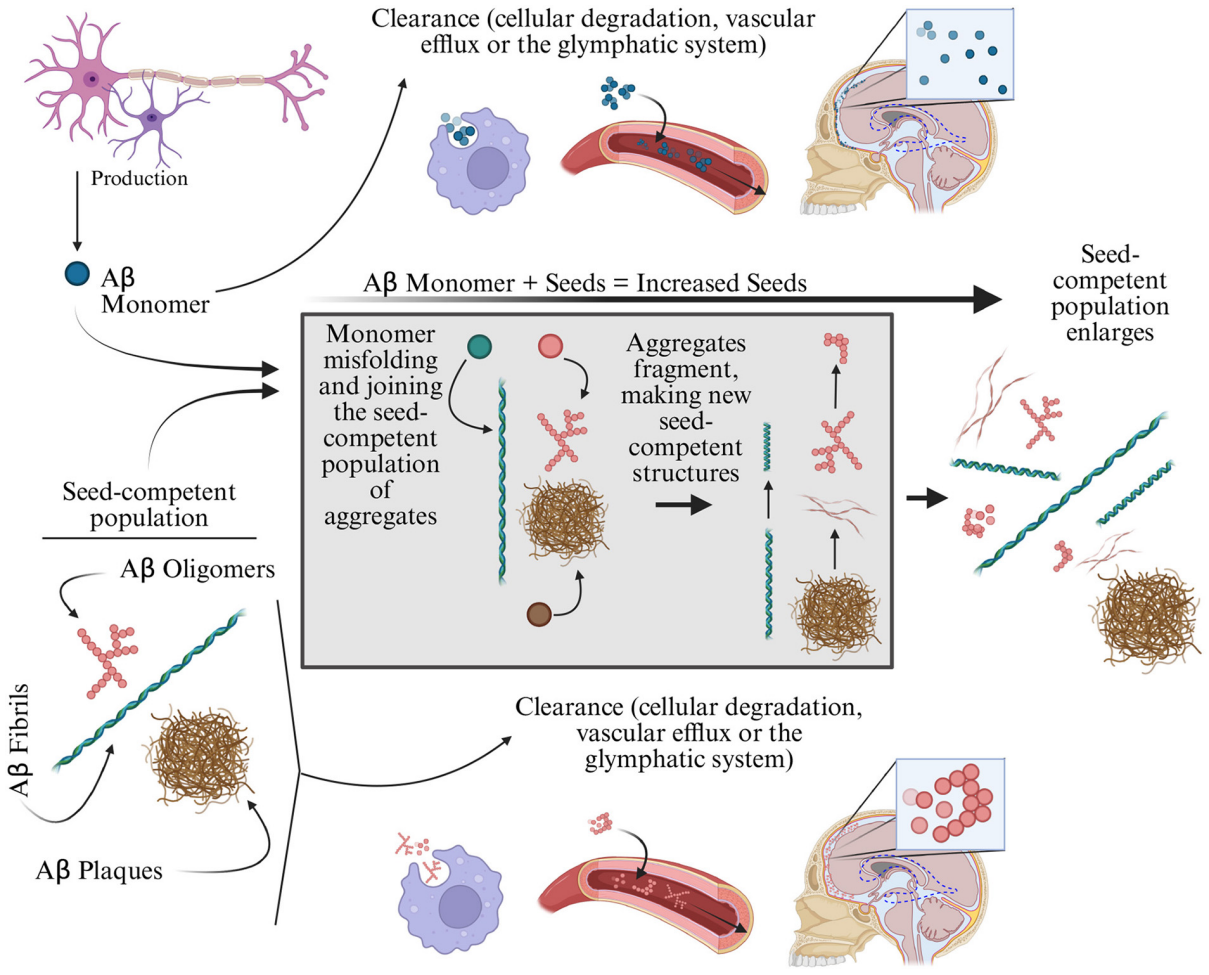
The Heterodimer-Homodimer model in AD simplifies complex dynamics. An Aβ monomer population (*p*, blue-green spheres) is produced (rate *a*) and cleared by cellular, vascular, or glymphatic mechanisms (rate *bp*, **top middle**). Aβ monomer in contact with the seed-competent population [$\hat{p}$, Aβ oligomers (composites of red spheres), fibrils (blue-green twisted ribbons), and plaques (brown hay-like masses) shown] misfolds, aggregates and fragments (gray box), creating new seed competent structures (rate $cp\hat{p}$). The seed competent population can also be cleared (rate $d\hat{p}$, **bottom middle**) by cellular, vascular or glymphatic means. τP kinetics produce the same set of equations.

Patients with plasma or serum folate ≤ 13.5nmol/L were 1.94 times more likely to be in the AD group than the control group (6 studies, *I*^2^ = 0.0%) while there was no evidence of a group preference if plasma/serum folate was above this cutoff. For the plasma/serum folate deficient group ( ≤ 13.5nmol/L), another meta-analysis (4 studies, *I*^2^ = 0.0%) found an 88% increase in (relative) incident AD risk. In addition, daily folate intake exceeding 400μg was associated with a 56% reduction in long-term AD risk (3 studies, *I*^2^ = 35.3%) and a 24% reduction in short-term AD risk (5 studies, *I*^2^ = 50%) [(132), Figures 3–5]. A recent meta-analysis looking at B-vitamins and incident dementia reinforced previous findings on folate but did not find an association between B_12_ or B_6_ (5 studies, *I*^2^ = 0%) and incident dementia risk [(133), Figure 3]. Favonoids were also examined in two current, large-scale meta-analyses but no significant association (5 studies, *I*^2^ = 73.3.6% and 3 studies, *I*^2^ = 0%) between flavonoid intake and incident AD risk was found in either case (130, 131) Finally, a recent meta-analysis has also considered the effects of vitamin D deficiency on AD risk. They found (6 studies, *I*^2^ = 63%) that serum/plasma Vitamin D levels below 25nmol/L increased long-term incident AD risk by 65% [(134), Figure 5], though it should be noted that the effects of vitamin D on AD risk may be mediated by ApoE ϵ4 status (135).

A potential microscale role for ω-3 fatty acids in AD was conjectured in one of Morris' early reviews (126). Around that time, epidemiological evidence was suggesting that dietary fish reduced AD risk; fish are rich in ω-3 polyunsaturated fats (PUFAs) like DHA, EPA and ALA. Sandra Kalmijn found a 60% reduction in AD risk with high fish consumption (≥ 18.5g/d) in a 1997 study [(136), Table 4]. Similar results from Pascale Barberger-Gateau and Morris quickly followed (137, 138), as did evidence, from Barberger-Gateau and Tina Huang, that the protective benefits of fish consumption on AD risk may be mediated by ApoE ϵ4 status (139, 140). These results led some to conclude that ω-3, abundant in fish, was reducing the risk of AD but, as Penny Dacks pointed out in 2013, this hypothesis had not yet been directly tested (141).

Contemporary evidence from recent meta-analyses has shed new, more nuanced, light on the hypothesis that ω-3 may reduce AD risk. Pooled evidence seems to suggest that high dietary fish intake may reduce incident AD risk (10 studies, *I*^2^ = 20%) by 20%. However, a subgroup analysis suggested that risk mitigation may vary depending on several factors like geographical region, the duration of the study and average participant age, etc. [(142), Figure 1, Table 2]. To further nuance the relation between ω-3 and AD risk, the recent results of a large US-based cohort study (*N* = 1,670) suggested that, despite substantial AD risk in the general ApoE ϵ4+ population, there was no evidence of difference in AD risk between ApoE ϵ4+ and ApoE ϵ4− individuals when ω-3 supplementation was both high and long-term [(143), Figures 4a, b]. This result suggests the possibility that the effects of ω-3 supplementation, on AD risk, may be mediated by ApoE ϵ4 status. A second large US-based cohort study (*N* = 1,135), which also included a meta-analysis, investigated this observation in more detail. This study first offered evidence that long-term ω-3 supplementation may reduce incident AD risk by at least 63% but that this effect may not be at all evident from measuring blood biomarkers and may be further mediated by sex, cognitive and ApoE ϵ4 status [(144), Tables 2, 3]. In particular, long term ω-3 supplementation reportedly reduced AD risk by 71% in the ApoE ϵ4+ group but no significant reduction was found for ApoE ϵ4− participants while none of the blood biomarkers (ω-3, DHA, ALA) were significantly associated with AD risk, regardless of ApoE ϵ4 status. A pursuant meta-analysis of the literature provided pooled effect estimates; studies adjusting for ApoE ϵ4 status reported that the risk of general cognitive decline was reduced by (8 studies, *I*^2^ = 65%) dietary ω-3 and by (9 studies, *I*^2^ = 42.4%) dietary DHA but not by general PUFA or EPA; this reduction vanished for studies not adjusting for ApoE ϵ4 status. For AD risk, the meta-analysis reported significant findings for a 24% reduction (6 studies, *I*^2^ = 56.9%) in AD risk by dietary DHA but not by other PUFA [(144), Figure 2, Supplementary Table 6]. Taken together, Morris' original conjecture for the role of ω-3 in AD may be correct but nuanced by the role of DHA, ApoE ϵ4 status and supplementation duration.

Fats other than ω-3s may influence AD risk at the microscale. Morris was the first to show that high levels of dietary saturated (SFAs) and trans fatty acids (TFAs) may increase AD risk by 2.2 and 2.4 times, respectively (145, 146). Ten years later, systematic reviews of SFAs and TFAs were available from studies conducted on large AD cohort data (CAIDE, Rotterdam, WHICAP and CHAP) (147, 148). High SFA consumption was associated with an increased MCI risk in the CAIDE cohort and an increased AD risk in the CHAP cohort [(147), Tables 1, 2]; SFAs were moderately associated with an elevated AD risk in WHICAP. CAIDE, CHAP and WHICAP showed evidence of ApoE ϵ4 status potentially mediating SFAs and AD risk or cognitive decline (147, 149).

A meta-analysis of the Rotterdam, CAIDE and CHAP studies found that SFAs may increase (3 studies, *I*^2^ = 0%) incident AD risk by 87% [(150), Table 3]. A second meta-analysis did not find evidence that dietary fat intkae, including SFA intake (6 studies, *I*^2^ = 57.6%), was related to AD risk [(151), Figure 2]. However, when the authors removed a single, highly heterogeneous Rotterdam cohort study from the meta-analysis, dietary SFAs were once more associated (5 studies, *I*^2^ = 0%) with an increase of AD risk at 32%. Results for TFAs remain mixed, showing a decrease in AD risk in the Rotterdam study with a potential increase found within the CHAP study (147, 148). Finally, levels of LDL and HDL cholesterol have now acquired an official “risk factor” status for dementia (3). A recent umbrella meta-analysis suggests evidence that high serum LDL levels may increase AD risk by 155% [(152), Figure 2, Table 2, Section 3.1]

Around the same time, a mendelian meta-analysis found that each 1mg/dL of total circulating cholesterol increased the incident risk of AD by 3% in ϵ3 relative to ϵ2 carriers and by 8% in ϵ4 relative to ϵ3 carriers. Each mg/dL reduction in circulating HDL-C was associated with a 130% increase in AD risk for ϵ4 vs. ϵ3 carriers but was not significant for ϵ3 vs. ϵ2 carriers (153). More recent large studies of high LDL and low HDL have focused on the risk of general dementia (154, 155) and the question of whether dietary levels of LDL and HDL may modify incident AD risk remains open.

The dietary microscale may also be related to the A/T/N biomarkers of AD (Section 2.2). A recent review (156) summarized the literature on microscale relationships observed in clinical trials. Low serum DHA levels were significantly related with brain Aβ PET, regardless of ApoE ϵ4 status, and serum DHA levels were positively correlated with both hippocampal and entorhinal regional brain volumes [(157), Figures 1B, 2A, B]. DHA supplementation may significantly reduce circulating Aβ42, but not Aβ40, levels and significantly increase brain clearance pathways [(158), Tables 3, 4]. Vitamin D supplementation may significantly reduce blood Aβ42, BACE1 and APP levels (both used in the making of Aβ) [(159), Figure 1, Table 4].

Three trials suggested that: serum DHA may be inversely correlated with brain amyloid PET, regardless of ApoE ϵ4 status [(157), Figure 1]; DHA supplementation may significantly decrease levels of blood Aβ_42_ [(158), Table 3]; and vitamin D supplementation may significantly reduce plasma Aβ_42_, alongside BACE1 and APP levels, [(159), Figure 1, Table 4] in patients with AD. Other studies showed that: blood LDL-c levels were positively correlated with Aβ PET (160); high blood LDL-c levels strengthened the correlation between Aβ and τP deposition (161); Aβ PET was negatively correlated with *B*_12_, Vitamin D, total ω-3 and ω-3, but not DHA, intake [(162), Table 2]. The latter study also investigated larger groupings of microscale nutrients, like “vitamin E with MUFA and PUFA,” and AD-associated brain regions. In AD regions: vitamin B and mineral intake was associated with increased cortical volume; vitamin E, MUFA, and PUFA intake was associated with increased metabolism; vitamin A, vitamin C, carotenoid, and fiber intake was associated with increased metabolism; vitamin *B*_12_, vitamin D, and zinc intake was associated with increased metabolism, increased cortical volume, and reduced Aβ; and saturated fats, trans fats, cholesterol and salt intake was associated with decreased metabolism and decreased cortical volume [(163), Tables 3–5]. This study raises the question of whether some microscale constituent interactions may be important for influencing AD biomarkers.

## 4 Mathematical network models of AD biomarkers

A scientific model is an accessible representation of a more complex system or process. Models are ubiquitous in the nutritional sciences (164); they are often used to learn, generate, test or predict hypotheses or outcomes for how a nutritional substance may positively or negatively impact human health (165, 166). A mathematical model (MM) is a scientific model that uses mathematics, instead of an organism or cells, as a means to quantify relationships of interest. Mechanistic MMs are frequently designed and used by nutritional scientists and obesity researchers, often working with mathematicians and engineers, to test the sufficiency, predictive power or range of possible measurements, or assumptions, on a system's outcomes. In this way, MMs can stand in for costly or ethically challenging experiments or be used to extrapolate experimental findings to other populations.

Nutritional scientists have been using MMs, of their own design, for several decades: energy balance mathematical models (EBMMs) (9). At the core of EBMMs is the energy balance principle *R* = *I*−*E*; *R*, *I*, and *E* are kcal/day stored, input and expended, respectively. The terms *R*, *I*, and *E* have been specialized across different models to study fat and lean body mass fluctuations, metabolic adaptations to bodyweight changes and the effect of diet composition, not just calories, on body weight and fat mass (167–170). MMs are now starting to appear in AD research. A new class of AD-related mathematical models (ADMMs) have emerged, to understand and predict A/T/N biomarker progression, which bear a close resemblance to the EBMMs used throughout the nutritional sciences. These new ADMMs are based on ordinary differential equations (ODEs), like EBMMs, but are posed on a network graph. These new network ADMMs (N-ADMMs) have been used to study and to predict A/T/N biomarker evolution (Section 2), but do not yet express the mechanisms that could link dietary patterns to that AD biomarker pathology. That current N-ADMMs do not express mechanisms related to dietary patterns is a significant gap. Closing this gap will enable novel, simulation-based AD-NAMs supporting research to reduce AD prevalence through dietary interventions.

This section introduces nutritional scientists and neuroscientists to the fundamental ideas underlying recent N-ADMMs and, specifically, their use to study AD biomarkers. This section may also be useful for mathematics and computer science research students who are not yet familiar with the practical aspects of modeling AD biomarkers mathematically on brain graphs. Sections 4.1–4.3 introduce the reader to the three essential building blocks of N-ADMMs: network ODEs, brain network graphs and the graph Laplacian. Sections 4.4–4.6 discusses the contemporary use of N-ADMMs to model A/T/N biomarker evolution. This section does not review other computational methods for AD research, such as the use of AI to study AD biomarker neuroimages. Instead, we focus on recent N-ADMMs for their relationships with EBMMs, their mechanistic interpretability, their ability to incorporate data and for their balance of spatial resolution with computational cost.

### 4.1 An accessible introduction to network differential equations

The use of mathematical models to study the brain dates back to the mid 20^th^ century (171, 172). MMs for AD research are more recent; significant computing power and advanced numerical methods have allowed complex, coupled systems of partial differential equations (PDEs) and high-spatial-resolution brain geometries to be used as MMs of AD pathology; these models can typically be solved in hours to days (173–177). These ADMMs have three drawbacks: they are often difficult to analyze mathematically, making computational simulations very important; however, they are too computationally expensive, especially for translational use; and they require highly specialized training to extract meshes from neuroimages, to design appropriate numerical methods and to implement the software for simulations. Conversely, EBMMs consist of a small number of ordinary differential equations (ODEs), can be solved in seconds or minutes and are amenable to mathematical analysis; their drawback is that they lack spatial resolution, an important factor for the evolution of A/T/N biomarkers, like Aβ and τP, in AD.

Network ODEs share in the advantages and limitations of traditional PDE and ODE models. They balance spatial resolution with computational cost and mathematical analyzability. Network ODE models have a three-part structure: (1) a graph *G*, which is often undirected; (2) a collection of ODEs associated to each vertex in the graph; and (3) a matrix, derived from the graph itself, that allows the ODEs defined at each graph vertex to communicate across the edges. For a simple example, consider the prototype ODE


$$\begin{array}{l} ẏ=\alpha y,\;y\left(0\right)=y_{0}, \end{array}$$ (4.1.A)

where α and *y*_0_ are pre-selected real numbers. The solution to Equation 4.1.A is $y\left(t\right)=y_{0}e^{\alpha t}$. Let *G* be a graph with two vertices, *v*_1_ and *v*_2_, and one edge connecting them. Consider *y*_1_ defined at *v*_1_ and *y*_2_ defined at *v*_2_ by Equation 4.1.A as


$$\begin{array}{l} {ẏ}_{1}=\alpha_{1}y_{1},\;{ẏ}_{2}=\alpha_{2}y_{2}, \end{array}$$ (4.1.B)

with *y*_1_(0), *y*_2_(0), α_1_ and α_2_ given. Equation 4.1.B can be made into a network ODE by introducing one or more *network communication matrices*. A network communication matrix is any matrix derived from the connectivity structure of the graph *G*; a simple example is the graph adjacency matrix. For our two-vertex graph *G* and ODE system (Equation 4.1.B), the adjacency matrix, *A*, and system state vector, **y**, are


$$A=\left[\begin{array}{cc} 0 & 1\\ 1 & 0 \end{array}\right],\;\text{y}\left(t\right)=\left[\begin{array}{c} y_{1}\left(t\right)\\ y_{2}\left(t\right) \end{array}\right].$$

Using *A* and **y**, above, the network ODE system can be expressed using either of the equivalent equations


$$\begin{array}{l} \dot{\text{y}}=A\text{y}+\left[\begin{array}{c} \alpha_{1}y_{1}\\ \alpha_{2}y_{2} \end{array}\right],\;\text{or}\;{\dot{\text{y}}}_{k}=\overset{2}{\underset{j=1}{\sum }}A_{kj}y_{j}+\alpha_{k}y_{k},\text{for}k=1,2 \end{array}$$ (4.1.C)

Given the choices above, Equation 4.1.C has only two equations. Written explicitly, these are


$$\begin{array}{l} \dot{y_{1}}=y_{2}+\alpha_{1}y_{1},\;\dot{y_{2}}=y_{1}+\alpha_{2}y_{2}. \end{array}$$ (4.1.D)

Equation 4.1.C, or equivalently Equation 4.1.D, show that *y*_1_ changes based on the value of *y*_2_ and vice versa, reflecting the connectivity of the graph *G*. This example demonstrates how network ODEs add spatial detail, through network communication matrices derived from *G*, while still reflecting the mathematical structure and lower computational complexity of the prototype ODE system (Equation 4.1.A) defined at each vertex, thus balancing the analytic potential, spatial resolution and computational cost of the resulting model.

### 4.2 The brain's structural connectome is a graph

In neuroscience, the term “connectome” refers to a connectivity graph between brain regions; a *functional connectome* encodes brain regions that activate together whereas a *structural connectome* shows how anatomical brain regions are connected by white matter projections (178). Structural connectomes (Figure 3) start with a patient MRI that includes T1 and diffusion weighted (DW) sequences. Second, the T1 image is segmented into anatomical regions; these regions will be the conntectome graph's vertices. Third, the segmented results and DW image data are processed by a tractography method determining which regions are connected by axonal projections; this step produces the connectome graph's edges (179, 180). Several software packages are now available that handle steps two and three (181–184) and have become an indispensable part of ADMMs based on network ODEs.

### 4.3 The graph Laplacian models diffusion on a graph

This section defines the graph Laplacian, a network communication matrix that models diffusion on an undirected graph *G*; in ADMMs, *G* is typically a structural connectome (Figure 3). Suppose the undirected graph *G* has *N* vertices labeled from *v*_1_ to *v*_*N*_. The adjacency matrix of *G* is defined as


$$A_{ij}=\{\begin{array}{cc} 1 & \text{if }v_{\text{i}}\text{ is connected to vertex }v_{\text{j}}\\ 0 & \text{otherwise} \end{array}$$ (4.3.A)

Using *A*, we define a diagonal matrix *D* with *i*^th^ diagonal entry *D*_*ii*_ determined by summing across the *i*^th^ row of *A* as:


$$\begin{array}{l} D_{ii}=\overset{N}{\underset{j=1}{\sum }}A_{ij}. \end{array}$$ (4.3.B)

The graph Laplacian of *G* is defined by


$$L_{ij}=\{\begin{array}{cc} D_{ii} & \text{when }i=j\\ -A_{ij} & \text{when }i\neq j \end{array}.$$ (4.3.C)

With an adjacency matrix defined by Equations 4.3.A, Equations 4.3.C amounts to *L*_*ij*_ being −1 when *i*≠*j* and *L*_*ii*_ is the sum of the entries in the *i*^th^ row of *A*. Intuitively, the *i*^th^ diagonal entry of *L* contributes to flow into graph vertex *v*_*i*_ while the off-diagonal entries contribute to flow out of *v*_*i*_. Simple diffusion on a graph *G* is then defined by either of the equivalent equations


$$\begin{array}{l} \dot{\text{y}}=-L\text{y},\;\text{or}\;{\dot{\text{y}}}_{k}=-\overset{N}{\underset{j=1}{\sum }}L_{kj}y_{j}, \end{array}$$ (4.3.D)

with an initial vector **y**(0) = **y**_0_ of non-negative entries. Since −*L*, in Equation 4.3.D, is derived from the connectivity of *G*, it is a network communication matrix for network ODE systems. In practice, brain connectomes are weighted graphs. A weighted graph has a positive weight *w*_*ij*_>0 associated to the edge connecting vertex *v*_*i*_ to vertex *v*_*j*_, indicating the strength of the edge connection. For a weighted graph *G*, the weighted adjacency matrix Â is


$${\hat{A}}_{ij}=\{\begin{array}{cc} w_{ij} & \text{if }v_{\text{i}}\text{ is connected to vertex }v_{\text{j}}\\ 0 & \text{otherwise} \end{array},$$

and the weighted diagonal matrix and weighted graph Laplacian follow from Equations 4.5.B, 4.3.C, using Â instead of *A*.

### 4.4 The first network ADMM was simple graph diffusion

The simplest network ADMM (N-ADMM) was developed by Ashish Raj in 2012 to study hypometabolism and atrophy due to Aβ or τP in AD (185, 186). This model assumed: a weighted structural connectome brain graph (Figure 3) was available; that Aβ or τP was correlated with hypometabolism and atrophy; and the spread of Aβ or τP was governed by simple graph diffusion as in Equation 4.3.D. The simple network ADMM was


$$\begin{array}{l} \dot{\text{p}}=-L\text{p},\;\text{with}\;\text{p}\left(0\right)={\text{p}}_{0}\;\text{and where}\;\text{p}\left(t\right)=\left[\begin{array}{c} p_{1}\left(t\right)\\ p_{2}\left(t\right)\\ \vdots \\ p_{N}\left(t\right) \end{array}\right], \end{array}$$ (4.4.A)

where *p*_*k*_(*t*) signifies Aβ or τP in brain region *v*_*k*_; the authors used the direct solution of Equation 4.4.A to derive an equation for hypometabolism and atrophy that could be directly computed [(185), Equation 6; (186), Equation 5]; they compared their predictions to data, marking the first validation of an N-ADMM. Raj and colleagues applied (Equation 4.4.A) to study other neurodegenerative diseases (187, 188). However, simple graph diffusion conserves misfolded protein mass and eventually settles into a uniform steady state. Thus, simple diffusion cannot model two important mechanisms in AD biomarker evolution: brain clearance and the prion-like hypothesis (Sections 2.3.2, 2.3.3). To address this, N-ADMMs would need an extension to account for the creation and removal of Aβ and τP biomarker mass.

### 4.5 Network ADMMs are based on a mass balance principle

Like the energy balance principle, *R* = *I*−*E* of EBMMs, ADMMs use a mass (or concentration) balance principle *B* = *P*−*C* where *B* is the rate that the mass (concentration) of a biomarker, like Aβ or τP, appears, *P* is the rate of biomarker mass (concentration) production and *C* is the rate of biomarker mass (concentration) clearance from the brain. The ODE form is Ḃ = *P*−*C* where *B* is now the biomarker mass (concentration). In an N-ADMM, there is one balance equation per brain region; since Aβ and τP can move from one brain region to another via white matter connectivity (graph edges), the balance principle in brain region *v*_*k*_ of the graph can be specialized to


$$\begin{array}{l} {Ḃ}_{k}=\left(W_{k}^{\text{in}}-W_{k}^{\text{out}}\right)+{\hat{P}}_{k}-{Ĉ}_{k}, \end{array}$$ (4.5.A)

where $W_{k}^{\text{in}}$ and $W_{k}^{\text{out}}$ are the incoming and outgoing rates of biomarker mass (concentration) via white matter (axon bundle) connections, and ${\hat{P}}_{k}$ and Ĉ_*k*_ represent the endogenous rates of biomarker production and clearance within brain region *v*_*k*_. Comparing with the balance principle *B* = *P*−*C*, the production (*P*) and clearance (*C*) rates of biomarker in each region is $P=W_{k}^{\text{in}}+{\hat{P}}_{k}$ and $C=W_{k}^{\text{out}}+{Ĉ}_{k}$; we drop the circumflex for simplicity. Currently, most N-ADMMs assume


$$\begin{array}{l} W_{k}^{\text{in}}-W_{k}^{\text{out}}=\overset{N}{\underset{j=1}{\sum }}A_{kj}\left(B_{j}-B_{k}\right), \end{array}$$ (4.5.B)

where *A*_*kj*_ is the *k*^th^ row and *j*^th^ column of the graph's (weighted) adjacency matrix. Equation 4.5.B is mathematically equivalent to using the negative (weighted) graph Laplacian, −*L*, as the network communication matrix. Thus, in general, the majority of N-ADMMs can be written in a vector form, like Equation 4.5.C, as


$$\begin{array}{l} {\dot{\text{B}}}_{k}=-\rho \overset{N}{\underset{j=1}{\sum }}L_{kj}B_{j}+{\text{p}}_{k}-{\text{C}}_{k}, \end{array}$$ (4.5.C)

where ρ>0 mediates the graph Laplacian diffusion (communication) speed and both **P**_*k*_ and **C**_*k*_ are typically defined by the same set of equations for every vertex *v*_*k*_, just as in Equation 4.5.C. Note that Equation 4.5.C satisfies this condition, where **P**_*k*_ = **C**_*k*_ = 0 for every brain region *v*_*k*_.

### 4.6 Contemporary ADMMs model prion-like AD biomarker evolution

Two watershed interdisciplinary studies were published in 2019; a computational study of prion-like spreading in neurodegenerative diseases (177); and, motivated by the previous work, the first N-ADMMs, all of which used a brain structural connectome graph (Figure 3), to incorporate the prion-like hypothesis for Aβ and τP in AD (189). The second work adapted three MMs from other fields to N-ADMMs: the Smoluchowski model, describing the kinetics of fragmenting and aggregating particles; the Heterodimer-Homodimer (HH) model used to studying prion protein (PrP); and the Fisher-Kolmogorov (FK) population model (189). The current state-of-the-art in network ADMMs can be mostly understood by examining (Equation 4.5.C) for the HH and FK models; for the Smoluchowski model, see Brennan and Goriely (190), Fornari et al. (189), and Thompson et al. (191).

Network ADMMs must explain observed AD biomarker data (72–74) and have prognostic capacity even when imaging data are scarce. To meet these challenges, Ellen Kuhl, Alain Goriely and colleagues proposed the FK network ADMM, defined by choosing


$${\text{p}}_{k}=\alpha {\text{p}}_{k},\;{\text{C}}_{k}=\alpha {\text{p}}_{k}^{2},$$

where α>0 is constant and **p**, instead of **B** in Equation 4.5.C, signifies Aβ or τP. This quadratic model expresses an initial exponential growth of misfolded and aggregated Aβ, followed by a linear growth phase which then reaches a plateau in each brain region *v*_*k*_ (Figure 4); this growth phenomenon was observed by Clifford Jack in patient Aβ image data (73). The normalized FK network model is


$$\begin{array}{l} {\dot{\text{p}}}_{k}=-\rho \overset{N}{\underset{j=1}{\sum }}L_{kj}p_{j}+\alpha p_{k}\left(1-p_{k}\right). \end{array}$$ (4.6.A)

Goriely, Kuhl and colleagues demonstrated the potential of Equation 4.6.A in data-driven AD research. They showed: that it supports the anisotropic axonal spreading hypothesis and not isotropic diffusion (192); that it can reproduce a wide array of τP staging patterns, including Braak staging (193); and that it can be used to infer patient parameters for τP spreading and atrophy rates (194–196). They also used a variant of Equation 4.6.A to test the hypothesis that brain clearance can significantly perturb the trajectory of τP pathology in AD (197).

The second fundamental N-ADMM is the HH model (Figure 5), which has a normal and a seed-competent (Aβ or τP) population at each vertex (198); the term “seed-competent” refers to a fibril, plaque or suitable oligomer that can initiate the misfolding and aggregation of another protein population. The prototypical form of the HH model is


$$\begin{array}{l} ṗ=a-bp-cp\hat{p} \end{array}$$ (4.6.Ba)

$$\begin{array}{l} \dot{\hat{p}}=-d\hat{p}+cp\hat{p}, \end{array}$$ (4.6.Bb)

where *p* and $\hat{p}$ are the normal Aβ (respectively τP) and seed-competent Aβ (respectively τP) population, *a*>0 and *b*>0 are the Aβ monomer production and clearance rates, *d*>0 is the clearance rate of seed-competent Aβ and *c*≥0 is the rate that new seed-competent Aβ (respectively τP) is formed from Aβ monomer (τP) and seed-competent Aβ (respectively τP). Equation 4.6.B can be lifted to an N-ADMM model by choosing


$$\begin{array}{ll} P_{k}^{\text{normal}}=a & C_{k}^{\text{normal}}=bp_{k}+cp_{k}{\hat{p}}_{k}\\ P_{k}^{\text{agg}}=cp_{k}{\hat{p}}_{k} & C_{k}^{\text{agg}}=d{\hat{p}}_{k} \end{array}$$

in Equation 4.5.C. The full expression is


$$\begin{array}{l} {\dot{\text{p}}}_{k}=-\rho \overset{N}{\underset{j=1}{\sum }}L_{kj}p_{j}+a-bp_{k}-cp_{k}{\hat{p}}_{k} \end{array}$$ (4.6.Ca)

$$\begin{array}{l} \dot{\hat{\text{p}}}=-\rho \overset{N}{\underset{j=1}{\sum }}L_{kj}{\hat{p}}_{j}-d{\hat{p}}_{k}+cp_{k}{\hat{p}}_{k}. \end{array}$$ (4.6.Cb)

Though more complex than Equations 4.6.A, 0.0.O is more physiologically descriptive; both Aβ and τP start as physiological monomers (**p**) and become toxic aggregates ($\hat{\text{p}}$) after associating with other toxic aggregates. The toxic aggregate population increases to a plateau (189), just as Equation 4.6.A does, in accordance with Aβ imaging data (73). Whereas having only two parameters gives Equation 4.6.A a data-fitting advantage, the increased expressiveness of Equation 4.6.C is often more suitable for investigating mechanistic AD phenomena, especially those observed *in vitro* or in animal models. Toward this end, Goriely and colleagues used the HH model to study a number of AD phenomena, including: interactions between Aβ and τP (199); the coupled nature of neuronal activity, Aβ and τP pathology (200, 201); and feedback between cerebrovascular integrity and Aβ spreading in AD (202).

Starting with the pioneering work of Raj et al. (185, 186) and Pandya et al. (187, 188) and continuing with a multitude of breakthrough contributions from Fornari et al. (189), Weickenmeier et al. (177), Brennan and Goriely (190), Thompson et al. (191), Schafer et al. (192), Putra et al. (193), Chaggar et al. (194), Schafer et al. (195), Schafer et al. (196), Brennan et al. (197), Thompson et al. (199), Alexandersen et al. (200, 201), and Ahern et al. (202), we have seen enormous contemporary progress in N-ADMMs. N-ADMMs balance computational cost, spatial expressiveness and relatively approachable mathematical analysis. Similar to EBMMs, N-ADMMs are rooted in a conservation principle (Equation 4.5.A). N-ADMMs use connectome graphs constructed from patient neuroimages; they can fit patient A/T/N biomarker data for prognoses and be used to explore mechanistic hypotheses. However, N-ADMMs do not yet express the mechanisms to connect them to modifiable risk factors mediated by diet. Linking diet to N-ADMMs is an interdisciplinary frontier in AD and nutritional science research.

## 5 Discussion

The question of “*how modifiable risk factors affect AD biomarker evolution*” is of contemporary importance. Dietary patterns are mutable behaviors that are related to a third of the modifiable risk factors identified for AD (2, 3). The World Health Organization and the National Institutes of Health are now openly calling for the development of innovative *new approach methodologies* to aid in the research effort for human diseases like AD and to ease the burden of animal testing, whenever possible. At the same time, computational AD-NAMs, based on N-ADMMs, are already being used (Section 4) to study and to simulate the evolution of important AD biomarkers within the A/T/N framework and to make predictions from neuroimaging data (Section 2).

A significant gap exists between current N-ADMMs and the ability to use them as AD-NAMs to study how modifiable risk factors, like dietary patterns, affect AD biomarker evolution. A primary challenge in closing the gap between dietary patterns and N-ADMMs is the need for highly interdisciplinary collaborations between nutritional scientists, neuroscientists, computer scientists and mathematicians. These collaborations will require a common historical and contemporary foundation; to our knowledge, no accessible interdisciplinary foundation exists in the literature. This narrative review has provided that foundation in three parts: the fundamentals and contextual significance of AD A/T/N biomarker pathology (Section 2); a historical and contemporary account that dietary patterns may influence AD risk and AD pathology across three different scales of dietary patterns (Section 3); and an introduction to the state-of-the-art in N-ADMMs, a foundation for mechanistic, simulation-based AD-NAMs (Section 4).

There are two objectives for moving forward in the development of novel AD-NAMs, from our current foundation of N-ADMMs, for the study of how diet may help to prevent AD. First, research studies tracking, or developing, AD biomarkers should incorporate assessments of dietary patterns (Section 5.1). Longitudinal neuroimaging data is already being used with contemporary N-ADMMs to make predictions (192, 195, 196) but data on dietary patterns are missing from the majority of these AD studies. Tracking dietary data in longitudinal AD studies will allow newly-developed simulation-based AD-NAMs to incorporate, and learn from, that data to make predictions or to study mechanisms. Second, ADMMs should link evidence from the three dietary scales to AD biomarker evolution and to incident AD risk (Section 5.2). Though it remains an open question as to what, specifically, the mechanisms that link dietary patterns to AD biomarkers may be, we propose that extending the current N-ADMMs to include the effects of *oxidative stress, neuroinflammation*, and *insulin resistance* will serve as effective starting points for this effort. Both of these objectives are opportunities for novel research at the intersection of diet and AD, charting a path forward for novel, simulation-based AD-NAMs in AD prevention research.

### 5.1 Improving dietary research in AD

Despite decades of hypotheses for AD pathogenesis and progression, no clear mechanistic consensus has emerged. The current view is that 14 modifiable factors account for up to 45% of the risk in developing AD; one third of these are mediated by diet. Despite this strong potential, contemporary evidence for the effects of dietary patterns on AD are only partially conclusive (3). This may be due to known challenges with dietary recall assessments, but may also arise due to obstacles within the dietary patterns themselves. First, macroscale dietary patterns can vary in constituents, making them challenging to link to AD mechanisms; they often need high adherence, for longer terms, to alter incident AD risk. Second, microscale constituents must be carefully considered in AD studies; they can exhibit dose-dependent responses, interactions with other microscale constituents and mediation by ApoE ϵ4 allele status. The macroscale DII/E-DII dietary pattern sits between the macro- and microscale; it offers a connection to a specific AD-related mechanism while allowing for some variation in dietary constituents. The obstacle of the DII/E-DII is that it was non-trivial to construct and took considerable time and effort to validate. We argue that addressing the following factors will help improve dietary research in AD: uncoupling the clinical-pathologic definition of AD; and including assessments of dietary intake in AD biomarker studies.

AD has long been viewed as a combined clinical-pathologic entity with post-mortem diagnostic confirmation (17). This dual view ties biomarker pathology, primarily Aβ and τP, to a clinical presentation confounded by comorbidities, cognitive reserve or other factors. Studies assessing interventions modifying neuropathic changes are encumbered by this dual definition; was AD affected if an intervention reduced neuropathology without altering clinical symptoms, or vice versa? A new definition, predicated on A/T/N biomarkers, disentangles AD from clinical stage while presenting a second rating system, for those stages, that compliments the biomarker-defined AD status [(25), Tables 4–7]. We propose that dietary studies of AD should adopt this uncoupled framework, enabling a clear separation of biological AD from clinical AD. Direct opportunities for further research include designing new studies that consider AD and diet, from this view, in addition to revisiting past cohort studies, where data are available (CAIDE, WHICAP, CHAP, etc.), and consider them in terms of these new criteria.

Dietary research in AD will be improved when AD biomarker research begins including dietary assessments alongside the full suite of, at least, the Core 1 A/T/N biomarkers [(25), Table 1] in addition to their other assessments. A number of large, well-known longitudinal studies have collected AD biomarkers and clinical measures. Examples include the Alzheimer's Disease Neuroimaging Initiative (ADNI), the Mayo Clinic Study of Aging (MCSA), the Swedish Biofinder Study (SBF), and the UK Biobank (UKB) (203–206). The ADNI, SBF, and UKB are the three largest contemporary AD biomarker studies in the world with ADNI being the most accessible. However, the SBF is the only one of the three with the requisite data to examine the effects of a more comprehensive dietary intake within the A/T/N framework. We suggest that, even for smaller scale studies, AD biomarker researchers adopt standardized, or enhanced, dietary intake assessments into their study. Standard intake tools are entirely non-invasive and include 24-h recall, food records, food frequency questionnaires, and screening tools (207). To enhance these tools, researchers may also consider screening blood and urine samples for biomarkers of food intake (BFI); BFIs can help to correct recollection errors encountered when using standard intake tools (208). Finally, including dietary information in longitudinal studies of AD biomarkers, especially for subjects recruited from previous longitudinal dietary studies, will ease the burden on developing novel mesoscale dietary patterns, like the DII/E-DII, that target specific AD-related mechanisms, like inflammation, oxidative stress, or insulin resistance. The a priori combination of diet and AD biomarkers will enable a forward analysis of dietary contribution and partially alleviate the otherwise pursuant necessity of a lengthy validation period.

### 5.2 Toward simulation-based AD-NAMs for dietary AD research

The role of diet in AD remains debated and traditional research methodologies face practical challenges, at each dietary scale, that go beyond their steep human, animal and economic costs. Variations in an individual's dietary patterns and the duration of adherence to a dietary pattern presents difficulties at the dietary macroscale. At the dietary mesoscale, a lack of dietary patterns linking diet to AD-related mechanisms, other than inflammation, is a barrier to progress. Interactions between different dietary components, between dietary components and ApoE ϵ4 allele status and transport of dietary components across the blood brain barrier all impose significant complexity at the microscale. Across all dietary scales, disharmony in study design, study protocols, measured variables, control variables, and a general lack of dietary assessments in AD biomarker and clinical research suggest a continued uphill battle toward strong evidence and a clearly, prevailing consensus. To move forward, we must either gather increasingly vast amounts of data, paying ever more human, animal and economic cost, or we must, somehow, incorporate new approaches. To this end, AD-NAMs, such as those based on mathematical models and computer simulations, could prove to be effective research tools to further explore these questions while reducing the burden on human, animal and economic resources.

Models simplify complex research landscapes and MMs, like their animal counterparts, have long been a tool to investigate the potential role, or expressive ability, of central mechanistic factors. The use of MMs and computing in, or related to, nutritional sciences is not new (168–170, 209). MMs of prion-like kinetics, as seen in AD, are also not new (198); neither is the notion that diet affects AD (80). N-ADMMs that express the prion-like hypothesis of AD are, however, recent and were first co-designed by a collaborative team of neuroscientists, mathematicians and engineers (189). N-ADMMs are mass balance models. Their general similarity to energy balance models places the nutritional sciences as a leading force in the development of the N-ADMMs that will serve as simulation-based AD-NAMs in preventative AD research. These types of AD-NAMs can help to improve predictive accuracy, reduce research costs, reduce the general reliance on animal models and potentially be integrated into clinical trials that look toward diet as a means to lower AD prevalence. In this direction, we foresee that mechanisms related both to diet and to neuronal stress, the response of neurons to exogenous or endogenous perturbations to cellular homeostasis, as promising starting points for providing this link; at a high level, these include: inflammation, oxidative stress and insulin resistance (90).

Inflammation, oxidative stress and insulin resistance are our suggested starting points for bridging dietary patterns and N-ADMMs. These three factors are at the intersection of human dietary studies with significant effects on incident AD risk and AD biomarkers (Section 3). At the macroscale (Section 3.1), the MED diet is rich in polyphenols, carotenoids, whole grains, fiber, and fish which reduce inflammation and oxidative stress (210, 211). Conversely, the WD shows increased inflammation and oxidative stress (210) while highly processed, fatty, and highly caloric foods, all hallmarks of the WD, are associated with brain insulin resistance (212). At the mesoscale (Section 3.2, the DII inflammatory score relates diet to inflammation, incident dementia and AD. At the microscale (Section 3.3), pooled evidence suggested that vitamins E, C, D, and folic acid may mediate AD risk; vitamins E, C, and folate all possess well-known antioxidant properties and vitamin D is a well-known anti-inflammatory. Vitamin D may also increase brain insulin sensitivity (213). Similarly, ω-3 fatty acids may be neuroprotective against, and mediate, neuroinflammation and it seems that the brain's access to DHA is may be by ApoE ϵ4 status (214–216). To study the implications of inflammation, oxidative stress and insulin resistance on AD biomarkers, N-ADMMs will need new expressions of the form (Equations 4.5.A, 4.5.B). Guided by nutritional scientists and neuroscientists, the correct populations, like Aβ, τP, inflammatory agents, reactive oxygen species, and antioxidants, will need to be determined; their interactions and spreading patterns will need to be modeled mathematically, studied, and validated against known AD characteristics. This is a challenging and long-term endeavor but the benefits may be significant. N-ADMMs provide a novel way to generate new research questions, explore existing hypotheses and to make predictions from patient data. N-ADMMs enhanced with dietary pattern-related mechanisms may form an effective foundation for novel, simulation-based AD-NAMs explore hypotheses, improve predictive accuracy, reduce research costs, reduce our reliance on animal testing and assist in the next generation of clinical trials aimed at the possibility that dietary patterns may be an effective intervention to help to reduce AD prevalence.

### 5.3 Limitations of AD-NAMs built on network mathematical models

Like any technology, network mathematical models have their limitations and these limitations will percolate through to any subsequent development, like AD-NAMs, built upon their foundation. Probably the most significant limitation of using the models of Section 4 to construct AD-NAMs is that the endeavor will require a significant upfront cost in fundamental interdisciplinary research effort. In particular, extending current N-ADMMs to incorporate dietary-relevant mechanisms, like oxidative stress, inflammation, and insulin resistance, will require guidance from nutritional scientists and neuroscientists as mathematicians or computer scientists work to determine mathematical formulations that capture the biological mechanisms within the language of network differential equations. This stands in sharp contrast to using a black-box machine learning method, like a simple deep neural network, to “just predict” outcomes from data. Black-box approaches can have a significantly lower up-front development cost but may have higher back end costs. They can requiring large amounts of data to accurately train, the resulting model not being interpretable, let alone amenable to mathematical analysis, and can be difficult to use for hypothesis testing involving questions about the potential effects of particular mechanisms or their interactions.

Working directly with extended N-ADMMs as a foundation for simulation-based AD-NAMs is potentially limited by two practical factors. First, brain graph networks are needed for these models. Existing graphs are available online [(193), Introduction], but should the need arise to construct patient-specific graphs, this can be a time consuming process. There are open source software tools available (217) to construct patient-specific brain networks, but even these tools require pre-processing steps for which a background in neuroimaging may be helpful. Thus, it is suggested to use readily available network brains graphs, at least in the preliminary stages of developing your N-ADMM for your downstream simulation-based AD-NAM. A second drawback is that solving the systems of Section 4, or their extended counterparts, requires software development. In particular, the authors are not aware of any “drag and drop” type of commercial solvers for this application that would make the process approachable to researchers without a computing background. However, there are a wealth of programmable commercial products and open source software libraries that can be used to solve large systems of differential equations quickly.

There are two further limitations to the N-ADMMs discussed in Section 4. First, they are deterministic. Interdisciplinary teams that wish to include random fluctuations alongside deterministic behavior in their AD-NAMs can consider, instead, extending the models of Section 4 to families of stochastic differential equations. These extensions also imply that solving the resulting N-ADMM, for simulation-based AD-NAMs will require more specialized stochastic differential equation solvers, though such solvers can be found in common commercial packages including Matlab, Mathematica and several open source libraries like Diffrax, Diffeqpy, PySDE, and DifferentialEquations.jl, among others. Second, teams that want to use data-driven AD-NAMs should keep in mind that N-ADMMs can quickly become complex. Recent work has demonstrated that statistical machine learning can be used with N-ADMMs to learn their parameters from neuroimaging data (192, 195, 196), but these authors used relatively simple, inferable N-ADMMs. Constructing separate N-ADMMs for inference and prediction versus those for hypothesis testing may be the best practice. This separation implies that simulation-based AD-NAMs should be designed to answer a specific set of research questions or to provide certain predictive capacities. Nevertheless, these complexities also suggest that developing novel N-ADMMs to enable simulation-based AD-NAMs is a interesting, rewarding and open field at the frontier of nutritionally based preventative AD research.

## References

[1] United Nations Department Department of Economic and Social Affairs Population Division. World Population Prospects 2022: Data Sources (UN DESA/POP/2022/DC/NO. 9). (2022). Available online at: https://www.population.un.org/wpp/Publications/Files/WPP2022_Data_Sources.pdf (Accessed March, 2024).

[2] Livingston G Huntley J Sommerlad A Ames D Ballard C Banerjee S . Demential prevention, intervention, and care: 2020 report of the Lancet Commission. Lancet. (2020) 396:413–46. doi: 10.1016/S0140-6736(20)30367-6 32738937 PMC7392084

[3] Livingston G Huntley J Liu KY Costafreda SG Selbæk G Alladi S . Dementia prevention, intervention, and care: 2024 report of the Lancet standing Commission. Lancet. (2024) 404:572–628. doi: 10.1016/S0140-6736(24)01296-0 39096926

[4] Dall T Reynolds R Chakrabarti R Ruttinger C Zareck P Parker O. The complexities of physician supply and demand: projections from 2021 to 2036. The Association of Americal Medical Colleges (2024). Available online at: https://www.aamc.org/media/75236/download?attachment (Accessed March, 2024).

[5] World Health Organization. Global Action Plan on the Public Health Response to Dementia 2017-2025. Report No. ISBN 978-92-4-151348-7 (2017).

[6] FDA. FDA Announces Plan to Phase Out Animal Testing Requirement for Monoclonal Antibodies and Other Drugs. (2025). Available online at: https://www.fda.gov/news-events/press-announcements/fda-announces-plan-phase-out-animal-testing-requirement-monoclonal-antibodies-and-other-drugs (Accessed October, 2025).

[7] FDA. New Approach Methods (NAMs). (2025). Available online at: https://www.fda.gov/food/toxicology-research/new-approach-methods-nams (Accessed October, 2025).

[8] NIH. NIH Funding Announcements to Align with NIH Initiative to Prioritize Human-based Research. (2025). Available online at: https://www.grants.nih.gov/news-events/nih-extramural-nexus-news/2025/07/nih-funding-announcements-to-align-with-nih-initiative-to-prioritize-human-based-research (Accessed October, 2025).

[9] Hall KD Farooqi IS Friedman JM Klein S Loos RJF Mangelsdorf DJ . The energy balance model of obesity: beyond calories in, calories out. Am J Clin Nutr. (2022) 115:1243–54. doi: 10.1093/ajcn/nqac031 35134825 PMC9071483

[10] Hippius H Neundörfer G. The discovery of Alzheimer's disease. Dialogues Clin Neurosci. (2003) 5:101–8. doi: 10.31887/DCNS.2003.5.1/hhippius 22034141 PMC3181715

[11] Alzheimer A Stelzmann R Schnitzlein H Murtagh F. An English translation of Alzheimer's 1907 paper “Über eine eigenartige Erkanku der Hirnride”. Clin Anat. (1995) 8:429–31. doi: 10.1002/ca.980080612 8713166

[12] Blessed G Tomlinson B Roth M. The association between quantitative measures of dementia and of senile change in the cerebral grey matter of elderly subjects. Br J Psychiatry. (1968) 114:797–811. doi: 10.1192/bjp.114.512.797 5662937

[13] Roth M Tomlinson B Blessed G. Correlation between scores for dementia and counts of ‘senile plaques' in cerebral grey matter of elderly subjects. Nature. (1966) 209:109–10. doi: 10.1038/209109a0 5927229

[14] Burns A Tomlinson B Mann DMA. Observations on the brains of demented old people. B.E. Tomlinson, G. Blessed and M. Roth, Journal of the Neurological Sciences (1970) 11, 205–242; (1968) 7, 331–356. Int J Geriatr Psychiatry. (1997) 12:785–790. doi: 10.1016/0022-510X(70)90063-8 9283922

[15] Wilcock GK Esiri MM. Plaques, tangles and dementia. A quantitative study. J Neurol Sci. (1982) 56:343–56. doi: 10.1016/0022-510X(82)90155-1 7175555

[16] Jack CR Jr Albert MS Knopman DS McKhann GM Sperling RA Carrillo MC . Introduction to the recommendations from the National Institue on Aging-Alzheimer's Association workgroups on diagnostic guidelines for Alzheimer's disease. Alzheimers Dement. (2011) 7:257–62. doi: 10.1016/j.jalz.2011.03.004 21514247 PMC3096735

[17] Jack CR Jr Bennett DA Blennow K Carrillo MC Dunn B Haeberlein SB . NIAA-AA research framework: toward a biological definition of Alzheimer's disease. Alzheimers Dement. (2018) 14:535–62. doi: 10.1016/j.jalz.2018.02.018 29653606 PMC5958625

[18] McKhann G Drachman D Folstein M Katzman R Price D Stadlan EM. Clinical diagnosis of Alzheimer's disease: report of the NINCDS-ADRDA Work Group under the auspices of Department of Health and Human Services Task Force on Alzheimer's Disease. Neurology. (1984) 34:939–44. doi: 10.1212/WNL.34.7.939 6610841

[19] American Psychiatric Association. Diagnostic and Statistical Manual of Mental Disorders (5th ed., text rev.). Washington, DC: American Psychiatric Association (2022). doi: 10.1176/appi.books.9780890425787

[20] DeTure M Dickson D. The neuropathological diagnosis of Alzheimer's disease. Mol Neurodegener. (2019) 14:32. doi: 10.1186/s13024-019-0333-5 31375134 PMC6679484

[21] Hardy J Higgins G. Alzheimer's disease: the amyloid cascade hypothesis. Science. (1992) 256:184–5. doi: 10.1126/science.1566067 1566067

[22] Hardy J Selkoe D. The amyloid hypothesis of Alzheimer's disease: progress and problems on the road to therapeutics. Science. (2002) 297:353–6. doi: 10.1126/science.1072994 12130773

[23] Goate A Hardy J. Twenty years of Alzheimer's disease-causing mutations. J Neurochem. (2011) 120:3–8. doi: 10.1111/j.1471-4159.2011.07575.x 22122678

[24] Jack CR Jr Bennett DA Blennow K Carrillo MC Feldman HH Frisoni GB . A/T/N: an unbiased descriptive classification scheme for Alzheimer's disease biomarkers. Neurology. (2016) 87:539–47. doi: 10.1212/WNL.0000000000002923 27371494 PMC4970664

[25] Jack CR Jr Andrews JS Beach TG Buracchio T Dunn B Graf A . Revised criteria for diagnosis and staging of Alzheimer's disease: Alzheimer's Association Workgroup. Alzheimers Dement. (2024) 20:5143–69. doi: 10.1002/alz.13859 38934362 PMC11350039

[26] Dawkins E Small D. Insights into the physiological function of the β-amyloid precursor protein: beyond Alzheimer's disease. J Neurochem. (2014) 129:756–69. doi: 10.1111/jnc.12675 24517464 PMC4314671

[27] Dunot J Ribera A Poushinha P Marie H. Spatiotemporal insights of APP function. Curr Opin Neurobiol. (2023) 82:102754. doi: 10.1016/j.conb.2023.102754 37542943

[28] Almeida Z Vaz D Brito R. Morphological and molecular profiling of amyloid-β species in Alzheimer's pathogenesis. Mol Neurobiol. (2025) 62:4391–419. doi: 10.1007/s12035-024-04543-4 39446217 PMC11880078

[29] Afram E Lauritzen I Bourgeois A El Manaa W Duplan E Chami M . The η-secretase-derived APP fragment ηCTF is localized in Golgi, endosomes and extracellular vesicles and contributes to Aβ production. Cell Mol Life Sci. (2023) 80:97. doi: 10.1007/s00018-023-04737-4 36930302 PMC10023608

[30] Andrew R Kellett K Thinakaran G Hooper N. A Greek Tragedy: the growing complexity of Alzheimer amyloid precursor protein proteolysis. J Biol Chem. (2016) 291:19235–44. doi: 10.1074/jbc.R116.746032 27474742 PMC5016663

[31] Haass C Kaether C Thinakaran G Sisodia S. Trafficking and proteolytic processing of APP. Cold Spring Harb Med. (2012) 2:a006270. doi: 10.1101/cshperspect.a006270 22553493 PMC3331683

[32] Burrinha T Almeida C. Aging impact on amyloid precursor protein neuronal trafficking. Curr Opin Neurobiol. (2022) 73:102524. doi: 10.1016/j.conb.2022.102524 35303572

[33] Hampel H Hardy J Blennow K Chen C Perry G Vergallo A . The amyloid-β pathway in Alzheimer's disease. Mol Psychiatry. (2021) 26:5481–503. doi: 10.1038/s41380-021-01249-0 34456336 PMC8758495

[34] Nunan J Small D. Regulation of APP cleavage by α-, β- and γ-secretases. FEBS Lett. (2000) 483:6–10. doi: 10.1016/S0014-5793(00)02076-7 11033346

[35] Yao Y Kang S Xia Y Wang ZH Liu X Ye K . A delta-secretase-truncated APP fragment activates CEBPB, mediating Alzheimer's disease pathologies. Brain. (2021) 144:1833–52. doi: 10.1093/brain/awab062 33880508 PMC8320270

[36] Zhang YW Thompson R Zhang H Xu H. APP processing in Alzheimer's disease. Mol Brain. (2011) 4:3. doi: 10.1186/1756-6606-4-3 21214928 PMC3022812

[37] Zhang Z Tian Y Ye K. δ-secretase in neurodegenerative diseases: mechanisms, regulators and therapeutic opportunities. Transl Neurodegener. (2020) 9:1. doi: 10.1186/s40035-019-0179-3 31911834 PMC6943888

[38] Limorenko G Lashuel H. Revisiting the grammar of Tau aggregation and pathology formation: how new insights from brain pathology are shaping how we study and target Tauopathies. Chem Soc Rev. (2022) 51:513–65. doi: 10.1039/D1CS00127B 34889934

[39] Barbier P Zejneli O Martinho M Lasorsa A Belle V Landrieu I . Role of tau as a microtubule-associated protein: structural and functional aspects. Front Aging Neurosci. (2019) 11:204. doi: 10.3389/fnagi.2019.00204 31447664 PMC6692637

[40] Qiang L Sun X Austin T Muralidharan H Jean D Baas P . Tau does not stabilize axonal microtubules but rather enables them to have long labile domains. Curr Biol. (2018) 28:2181–9. doi: 10.1016/j.cub.2018.05.045 30008334

[41] Alonso A Clerico J Li B Corbo C Alaniz M Iqbal K . Phosphorylation of Tau at Thr^2^12, Thr^2^31, and Ser^2^62 combined causes neurodegeneration. J Biol Chem. (2010) 285:30851–60. doi: 10.1074/jbc.M110.110957 20663882 PMC2945578

[42] Chakraborty P Ibanez de Opakua A Purslow J Fromm S Chatterjee D Zweckstetter M . GSK3β phosphorylation catalyzes the aggregation of tau into Alzheimer's disease-like filaments. Proc Natl Acad Sci. (2024) 121:e2414176121. doi: 10.1073/pnas.2414176121 39693350 PMC11670061

[43] Reyes-Pablo A Luna-Viramontes N Montiel-Sosa J Ontiveros-Torres M Garces-Ramirez L Luna-Munoz J . Vulerability of the entorhinal cortex II to neurodegeneration in Alzheimer's disease. Brain. (2025) 7:fcaf091. doi: 10.1093/braincomms/fcaf091 PMC11897590 40078869

[44] Wesseling H Mair W Kumar M Schlaffner C Tang S Steen J . Tau PTM profiles identify patient heterogeneity and stages of Alzheimer's disease. Cell. (2020) 183:1699–713. doi: 10.1016/j.cell.2020.10.029 33188775 PMC8168922

[45] Harper J Wong S Lansbury P. Observation of metastable Abeta amyloid protofibrils by atomic force microscopy. Chem Biol. (1997) 4:119–25. doi: 10.1016/S1074-5521(97)90255-6 9190286

[46] Cardenas-Aguayo M Gomez-Virgilio L DeRosa S Meraz-Rios M. The role of tau oligomers in the onset of Alzheimer's disease neuropathology. ACS Chem Neurosci. (2014) 5:1178–91. doi: 10.1021/cn500148z 25268947

[47] Rinauro D Chiti F Vendruscolo M Limbocker R. Misfolded protein oligomers: mechanisms of formation, cytotoxic effects, and pharmacological approaches against protein misfolding diseases. Mol Neurodegener. (2024) 19:20. doi: 10.1186/s13024-023-00651-2 38378578 PMC10877934

[48] Caughey B Lansbury P. Protofibrils, pores, fibrils, and neurodegeneration: separation the responsible protein aggregates from the innocent bystanders. Annu Rev Neurosci. (2003) 26:267–98. doi: 10.1146/annurev.neuro.26.010302.081142 12704221

[49] Goure W Krafft G Jerecic J Hefti F. Targeting the proper amyloid-beta neuronal toxics: a path forward for Alzheimer's disease immunotherapeutics. Alzheimers Res Ther. (2014) 6:42. doi: 10.1186/alzrt272 25045405 PMC4100318

[50] Hayden E Teplow D. Amyloid β-protein oligomers and Alzheimer's disease. Alzheimers Res Ther. (2013) 5:60. doi: 10.1186/alzrt226 24289820 PMC3978746

[51] Mrdenovic D Pieta I Nowakowski R Kutner W Lipkowski J Pieta P. Amyloid β interaction with model cell membranes - What are the toxicity-defining properties of amyloid β? Int J Biol Macromol. (2022) 200:520–31. doi: 10.1016/j.ijbiomac.2022.01.117 35074328

[52] Ono K Condron M Teplow D. Structure-neurotoxicity relationships of amyloid beta-protein oligomers. Proc Natl Acad Sci. (2009) 106:14745–50. doi: 10.1073/pnas.0905127106 19706468 PMC2736424

[53] Azargoonjahromi A. The duality of amyloid-β: its role in normal and Alzheimer's disease states. Mol Brain. (2024) 17:44. doi: 10.1186/s13041-024-01118-1 39020435 PMC11256416

[54] Gulisano W Melone M Ripoli C Tropea M Li Puma D Puzzo D . Neuromodulatory action of picomolar exracellular Aβ42 oligomers on presynaptic and postsynaptic mechanisms underlying synaptic function and memory. J Neurosci. (2019) 39:5986–6000. doi: 10.1523/JNEUROSCI.0163-19.2019 31127002 PMC6650983

[55] Kent S Spires-Jones T Durrant C. The physiological roles of tau and Aβ: implications for Alzheimer's disease pathology and therapeutics. Acta Neuropathol. (2020) 140:417–47. doi: 10.1007/s00401-020-02196-w 32728795 PMC7498448

[56] Hill E Karikari T Moffat K Richardson M Wall M. Introduction of tau oligomers into cortical neurons alters action potential dynamics and disrupts synaptic transmission and plasticity. eNeuro. (2019) 6:ENEURO.0166-19.2019. doi: 10.1523/ENEURO.0166-19.2019 31554666 PMC6794083

[57] Mawuenyega K Sigurdson W Ovod V Munsell L Kasten T Bateman R . Decreased clearance of CNS beta-amyloid in Alzheimer's disease. Science. (2010) 330:1774. doi: 10.1126/science.1197623 21148344 PMC3073454

[58] Nalivaeva N Turner A. Targeting amyloid clearance in Alzheimer's disease as a therapeutic strategy. Br J Pharmacol. (2019) 176:3447–63. doi: 10.1111/bph.14593 30710367 PMC6715594

[59] Patterson B Elbert D Mawuenyega K Kasten T Ovod V Bateman R . Age and amyloid effects on human central nervous system amyloid-beta kinetics. Ann Neurol. (2015) 78:439–53. doi: 10.1002/ana.24454 26040676 PMC4546566

[60] Hablitz L Nedergaard M. The glymphatic system: a novel component of fundamental neurobiology. J Neurosci. (2021) 41:7698–711. doi: 10.1523/JNEUROSCI.0619-21.2021 34526407 PMC8603752

[61] Hegde V Dhurandhar N Reddy PH. Hyperinsulinemia or insulin resistance: what impacts the progression of Alzheimer's disease? J Alzheimers Dis. (2019) 72:S71–9. doi: 10.3233/JAD-190808 31744006

[62] Lohela T Lilius T Nedergaard M. The glymphatic system: implications for drugs for central nervous system diseases. Nat Rev Drug Discov. (2022) 21:763–79. doi: 10.1038/s41573-022-00500-9 35948785

[63] Tarasoff-Conway J Carare R Osorio R Glodzik L Butler T de Leon M . Clearance systems in the brain-implications for Alzheimer disease. Nat Rev Neurol. (2015) 11:457–70. doi: 10.1038/nrneurol.2015.119 26195256 PMC4694579

[64] Ullah R Lee E. Advances in amyloid-β clearance in the brain and periphery: implications for neurodegenerative diseases. Exp Neurobiol. (2023) 32:216–46. doi: 10.5607/en23014 37749925 PMC10569141

[65] Wang L Sooram B Kumar R Schedin-Weiss S Tjernberg L Winblad B. Tau degradation in Alzheimer's disease: Mechanisms and therapeutic opportunities. Alzheimers Dement. (2025) 21:e70048. doi: 10.1002/alz.70048 40109019 PMC11923393

[66] Cohen F Pan K Huang Z Baldwin M Fletterick R Prusiner SB. Structural clues to prion replication. Science. (1994) 264:530–1. doi: 10.1126/science.7909169 7909169

[67] Fraser H Dickinson AG. Targeting of scrapie lesions and spread of agent via the retino-tectal projection. Brain Res. (1985) 346:32–41. doi: 10.1016/0006-8993(85)91091-1 4052769

[68] Prusiner SB. Novel proteinaceous infectious particles cause scrapie. Science. (1982) 216:136–44. doi: 10.1126/science.6801762 6801762

[69] Prusiner SB. Some speculations about prions, amyloid, and Alzheimer's disease. N Engl J Med. (1984) 310:661–3. doi: 10.1056/NEJM198403083101021 6363926

[70] Prusiner SB. Creutzfeldt-Jakob disease and scrapie prions. Alzheimer Dis Assoc Disord. (1989) 3:52–78. doi: 10.1097/00002093-198903010-00007 2568118

[71] Arnold S Hyman B Flory J Damasio A Van Hoesen G. The Topographical and neuroanatomical distribution of neurofibrillary tangles and neuritic plaques in the cerebral cortex of patients with Alzheimer's disease. Cereb Cortex. (1991) 1:103–16. doi: 10.1093/cercor/1.1.103 1822725

[72] Braak H Braak E. Neuropathological stageing of Alzheimer-related changes. Acta Neuropathol. (1991) 82:239–59. doi: 10.1007/BF00308809 1759558

[73] Jack C Wiste H Lesnick T Weigand S Knopman D Petersen R . Brain β-amyloid load approaches a plateau. Neurology. (2013) 80:890–6. doi: 10.1212/WNL.0b013e3182840bbe 23446680 PMC3653215

[74] Schöll M Lockhart S Schonhaut D O'Neil J Janabi M Jagust W . PET imaging of tau deposition in the aging human brain. Neuron. (2016) 89:971–982. doi: 10.1016/j.neuron.2016.01.028 26938442 PMC4779187

[75] Jaunmuktane Z Brandner S. Invited Review: the role of prion-like mechanisms in neurodegenerative diseases. Neuropathol Appl Neurobiol. (2020) 46:522–45. doi: 10.1111/nan.12592 31868945 PMC7687189

[76] Jucker M Walker L. Propagation and spread of pathogenic protein assemblies in neurodegenerative diseases. Nat Neurosci. (2018) 21:1341–9. doi: 10.1038/s41593-018-0238-6 30258241 PMC6375686

[77] Sanders D Kaufman S Holmes B Diamond M. Prions and protein assemblies that convey biological information in health and disease. Neuron. (2016) 89:433–48. doi: 10.1016/j.neuron.2016.01.026 26844828 PMC4748384

[78] Walker L. Prion-like mechanisms in Alzheimer disease. Handb Clin Neurol. (2018) 153:303–19. doi: 10.1016/B978-0-444-63945-5.00016-7 29887142 PMC6375694

[79] Hallinan G Vargas-Caballero M West J Deinhardt K. Tau misfolding efficiently propagates between individual intact hippocampal neurons. J Neurosci. (2019) 39:9623–32. doi: 10.1523/JNEUROSCI.1590-19.2019 31658988 PMC6880463

[80] Newman P. Could diet be one of the causal factors of Alzheimer's disease? Med Hypotheses. (1992) 39:123–6. doi: 10.1016/0306-9877(92)90169-D 1461171

[81] Grant W. Dietary links to Alzheimer's disease. Alz Dis Rev. (1997) 2:42–55. 10.3233/jad-1999-14-501 12214118

[82] Hendrie H Osuntokun B Hall K Ogunniyi A Hui S Musick B . Prevalence of Alzheimer's disease and dementia in two communities: Nigerian Africans and African Americans. Am J Psychiatry. (1995) 152:1485–92. doi: 10.1176/ajp.152.10.1485 7573588

[83] White L Petrovitch H Ross W Masaki K Abbott R Curb D . Prevalence of dementia in older Japanese-American men in Hawaii. JAMA. (1996) 276:955–60. doi: 10.1001/jama.1996.03540120033030 8805729

[84] Clemente-Suarez V Beltran-Velasco A Renodo-Florez L Martin-Rodriguez A Tornero-Aguilera J. Global impacts of western diet and its effects on metabolism and health: a narrative review. Nutrients. (2023) 15:2749. doi: 10.3390/nu15122749 37375654 PMC10302286

[85] Eaton SB Konner M. Paleolithic nutrition. A consideration of its nature and current implications. N Engl J Med. (1985) 312:283–9. doi: 10.1056/NEJM198501313120505 2981409

[86] Eaton SB Konner M Shostak M. Stone agers in the fast lane: chronic degenerative diseases in evolutionary perspective. Am J Med. (1988) 84:739–49. doi: 10.1016/0002-9343(88)90113-1 3135745

[87] Kopp W. How western diet and lifestyle drive the pandemic of obesity and civilization diseases. Diabetes Metab Syndr Obes. (2019) 12:2221–36. doi: 10.2147/DMSO.S216791 31695465 PMC6817492

[88] Loren C Boyd E Anthony S Neil M Staffan L Janette BM . Origins and evolution of the Western diet: health implications for the 21st century. AJCN. (2005) 81:341–54. doi: 10.1093/ajcn.81.2.341 15699220

[89] Molly F. ‘Evolutionary medicine' perspectives on Alzheimer's Disease: review and new directions. Ageing Res Rev. (2018) 47:140–8. doi: 10.1016/j.arr.2018.07.008 30059789 PMC6195455

[90] Decourt B D'Sourza G Shi J Ritter A Suano J Sabbagh M. The cause of Alzheimer's disease: the theory of multipathology convergence to chronic neuronal stress. Aging Dis. (2022) 13:37–60. doi: 10.14336/AD.2021.0529 35111361 PMC8782548

[91] Grant W. Dietary links to Alzheimer's disease: 1999 update. J Alzheimers Dis. (1999) 1:197–351. doi: 10.3233/JAD-1999-14-501 12214118

[92] Keys A Keys M. Eat Well and Stay Well the Mediterranean Way. New York, NY: Doubleday. (1975).

[93] Appel LJ Moore TJ Obarzanek E Vollmer W Svetkey L Karanja N . A clinical trial of the effects of dietary patterns on blood pressure. DASH Collaborative Research Group. N Engl J Med. (1997) 336:1117–11124. doi: 10.1056/NEJM199704173361601 9099655

[94] Morris M Tangney C Wang Y Sacks F Barnes L Bennett D . MIND diet slows cognitive decline with aging. Alzheimers Dement. (2015) 11:1015–22. doi: 10.1016/j.jalz.2015.04.011 26086182 PMC4581900

[95] van den Brink A Brouwer-Brolsma E Merendsen A van de Rest O. The mediterranean, dietary approaches to stop hypertension (DASH), and mediterranean-DASH intervention for neurodegenerative delay (MIND) diets are associated with less cognitive decline and a lower risk of Alzheimer's disease-a review. Adv Nutr. (2019) 10:1040–65. doi: 10.1093/advances/nmz054 31209456 PMC6855954

[96] Scarmeas N Stern Y Mayeux R Luchsinger J. Mediterranean diet, Alzheimer disease, and vascular mediation. Arch Neurol. (2006) 63:1709–17. doi: 10.1001/archneur.63.12.noc60109 17030648 PMC3024906

[97] Gu Y Luchsinger J Stern Y Scarmeas N. Mediterranean diet, inflammatory and metabolic biomarkers, and risk of Alzheimer's disease. J Alzheimers Dis. (2010) 22:483–92. doi: 10.3233/JAD-2010-100897 20847399 PMC3022949

[98] Morris M Tagney C Wang Y Sacks F Bennett D Aggarwal N. MIND diet associated with reduced incidence of Alzheimer's disease. Alzheimers Dement. (2015) 11:1007–14. doi: 10.1016/j.jalz.2014.11.009 25681666 PMC4532650

[99] Scarmeas N Stern Y Tang MX Mayeux R Luchsinger J. Mediterranean diet and risk for Alzheimer's disease. Ann Neurol. (2006) 59:912–21. doi: 10.1002/ana.20854 16622828 PMC3024594

[100] Scarmeas N Luchsinger J Schupf N Brickman A Cosentino S Tang M . Physical activity, diet, and risk of Alzheimer disease. JAMA. (2009) 302:627–37. doi: 10.1001/jama.2009.1144 19671904 PMC2765045

[101] Scarmeas N Stern Y Mayeux R Luchsinger J. Mediterranean diet and mild cognitive impairment. Arch Neurol. (2009) 66:216–25. doi: 10.1001/archneurol.2008.536 19204158 PMC2653223

[102] Chen H Dhana K Huang Y Tao Y Liu X Yuan C . Association of the mediterranean dietary approaches to stop hypertension intervention for neurodegenerative delay (MIND) diet with the risk of dementia. JAMA Psychiatry. (2023) 80:630–8. doi: 10.1001/jamapsychiatry.2023.0800 37133875 PMC10157510

[103] Khoshdooz S Bonyad A Bonyad R Khoshdooz P Jafari A Rahnemayan S . Role of dietary patterns in older adults with cognitive disorders: An umbrella review utilizing neuroimaging biomarkers. Neuroimage. (2024) 303:120935. doi: 10.1016/j.neuroimage.2024.120935 39547460

[104] Dhana K James B Agarwal P Aggarwal NT Cherian LJ Leurgans SE . MIND diet, common brain pathologies, and cognition in community-dwelling older adults. J Alzheimers Dis. (2021) 83:683–92. doi: 10.3233/JAD-210107 34334393 PMC8480203

[105] Wagner M Agarwal P Leurgans S Bennett D Schneider J Capuano A . The association of MIND diet with cognitive resilience to neuropathologies. Alzheimers Dement. (2023) 19:3644–53. doi: 10.1002/alz.12982 36855023 PMC10460833

[106] Agrawal P Leurgans S Agrawal S Cherian L James B Schneider J . Association of mediterranean-DASH intervention for neurodegenerative delay and mediterranean diets with Alzheimer disease pathology. Neurology. (2023) 100:e2259–68. doi: 10.1212/WNL.0000000000207176 36889921 PMC10259273

[107] Li J Capuano A Agarwal P Arvanitakis Z Wang Y Grodstein F . The MIND diet, brain transcriptomic alterations, and dementia. Alzheimers Dement. (2024) 20:5996–6007. doi: 10.1002/alz.14062 39129336 PMC11497672

[108] Cavicchia P Steck S Hurley T Hussey J Ma Y Ockene I . A new dietary inflammatory index predicts interval changes in serum high-sensitivity c-reactive protein. J Nutr. (2009) 139:2365–72. doi: 10.3945/jn.109.114025 19864399 PMC2777480

[109] Shivappa N Steck S Hurley T Hussey J Hébert J. Designing and developing a literature-derived, population-based dietary inflammatory index. Public Health Nutr. (2014) 17:1689–96. doi: 10.1017/S1368980013002115 23941862 PMC3925198

[110] Miller P Cross A Subar A Krebs-Smith S Park Y Reedy J . Comparison of 4 established DASH diet indexes: examining associations of index scores and colorectal cancer. Am J Clin Nutr. (2013) 98:794–803. doi: 10.3945/ajcn.113.063602 23864539 PMC3743737

[111] Hébert J Shivappa N Wirth M Hussey J Hurley T. Perspective: the dietary inflammatory index (DII)-lessons learned, improvements made, and future directions. AN/Adv Nutr. (2019) 10:185–195. doi: 10.1093/advances/nmy071 30615051 PMC6416047

[112] Jovanovic B Freels S VanEenwyk J. Nutrient density model revisited. Nutr Res. (1994) 14:765–74. doi: 10.1016/S0271-5317(05)80211-8

[113] Willett WC Howe GR Kushi LH. Adjustment for total energy intake in epidemiologic studies. AJCN. (1997) 65:1220S–8S. doi: 10.1093/ajcn/65.4.1220S 9094926

[114] Hayden K Beavers D Steck S Hebert J Tabung F Rapp S . The association between an inflammatory diet and global cognitive function and incident dementia in older women: the Women's Health Initiative Memory Study. Alzheimers Dement. (2017) 13:1187–96. doi: 10.1016/j.jalz.2017.04.004 28531379 PMC5909961

[115] Peng M Yuan S Lu D Ling Y Huang X Lyu J . Dietary Inflammatory index, genetic susceptibility and risk of incident dementia: a prospective cohort study for the UK biobank. J Neurol. (2024) 271:1286–96. doi: 10.1007/s00415-023-12065-7 37985486

[116] Shi Y Lin F Li Y Wang Y Chen X Cai G . Association of pro-inflammatory diet with increased risk of all-cause dementia and Alzheimer's dementia: a prospective study of 166,377 UK Biobank participants. BMC Med. (2023) 21:266. doi: 10.1186/s12916-023-02940-5 37480061 PMC10362711

[117] Zhang X Wang Y Liu W Wang T Wang L Xiao R . Diet quality, gut microbiota, and microRNAs associated with mild cognitive impairment in middle-aged and elderly Chinese population. Am J Clin Nutr. (2021) 114:429–40. doi: 10.1093/ajcn/nqab078 33871591

[118] Wang X Li T Li H Li D Wang X Xi Y . Association of dietary inflammatory potential with blood inflammation: the prospective markers on mild cognitive impairment. Nutrients. (2022) 14:2417. doi: 10.3390/nu14122417 35745147 PMC9229190

[119] Liu Q Zhou D Duan H Zhu Y Du Y Huang G . Association of dietary inflammatory index and leukocyte telomere length with mild cognitive impairment in Chinese older adults. Nutr Neurosci. (2023) 26:50–9. doi: 10.1080/1028415X.2021.2017660 34957928

[120] Dardiotis E Kosmidis M Yannakoulia M Hadjigeorgiou G Scarmeas N. The hellenic longitudinal investigation of aging and diet (HELIAD): rationale, study design, and cohort description. Neuroepidemiology. (2014) 43:9–14. doi: 10.1159/000362723 24993387

[121] Charisis S Ntanasi E Yannakoulia M Anastasiou C Kosmidis M Scarmeas N . Diet inflammatory index and dementia incidence: a population-based study. Neurology. (2021) 97:e2381–91. doi: 10.1212/WNL.0000000000012973 34759053 PMC8673721

[122] Ding T Aimaiti M Cui S Shen J Lu M Bian D . Meta-analysis of the association between dietary inflammatory index and cognitive health. Front Nutr. (2023) 10:1104255. doi: 10.3389/fnut.2023.1104255 37081917 PMC10111053

[123] Duggan M Butler L Peng Z Daya G Moghekar A Walker K . Plasma proteins related to inflammatory diet predict future cognitive impairment. Mol Psychiatry. (2023) 28:1599–609. doi: 10.1038/s41380-023-01975-7 36737481 PMC10208977

[124] Morris M Beckett L Scherr P Hebert L Bennett D Evans D . Vitamin E and Vitamin C supplement use and risk of incident Alzheimer disease. Alzheimer Dis Assoc Disord. (1998) 12:121–6. doi: 10.1097/00002093-199809000-00001 9772012

[125] Clarke R Smith D Jobst K Refsum H Sutton L Ueland PM. Folate, Vitamin B12, and Serum total homocysteine levels in confirmed Alzheimer disease. JAMA Neurol. (1998) 55:1449–55. doi: 10.1001/archneur.55.11.1449 9823829

[126] Morris MC. The role of nutrition in Alzheimer's disease: epidemiological evidence. Eur J Neurol. (2009) 16:1–7. doi: 10.1111/j.1468-1331.2009.02735.x 19703213 PMC3393525

[127] Lopes da Silva S Vellas B Elemans S Luchsinger J Kamphuis P Stijnen T . Plasma nutrient status of patients with Alzheimer's disease: Systematic review and meta-analysis. Alzheimers Dement. (2014) 10:485–502. doi: 10.1016/j.jalz.2013.05.1771 24144963

[128] Marwaha S Agarwal R Tripathi M Tripathi S. Unlocking the vitamin puzzle: investigating levels in people with Alzheimer's disease versus healthy controls through systematic review and network meta-analysis. J Hum Nutr Diet. (2025) 38:e70007. doi: 10.1111/jhn.70007 39763154

[129] Qu M Shi H Wang K Wang X Yu N Guo B. The associations of plasma/serum carotenoids with Alzheimer's disease: a systematic review and meta-analysis. J Alzheimers Dis. (2021) 82:1055–66. doi: 10.3233/JAD-210384 34151808

[130] Zhao R Han X Jiang S Zhao W Liu J You H . Association of dietary and supplement intake of antioxidants with risk of dementia: a meta-analysis of cohort studies. J Alzheimers Dis. (2024) 99:S35–50. doi: 10.3233/JAD-220909 36846999

[131] Zhou F Xie X Zhang H Liu T. Effect of antioxidant intake patterns on risks of dementia and cognitive decline. Eur Geriatr Med. (2023) 14:9–17. doi: 10.1007/s41999-022-00720-7 36445640

[132] Zhang X Bao G Liu D Yang Y Li X Wu Y . The association between Folate and Alzheimer's disease: a systematic review and meta-analysis. Front Neurosci. (2021) 15:661198. doi: 10.3389/fnins.2021.661198 33935641 PMC8079632

[133] Wang Z Zhu W Xing Y Jia J Tang Y. B vitamins and prevention of cognitive decline and incident dementia: a systematic review and meta-analysis. Nutr Rev. (2022) 80:931–49. doi: 10.1093/nutrit/nuab057 34432056

[134] Zhang XX Wang HR Wei M Hu YZ Sun HM Jia JJ . Association of Vitamin D levels with risk of cognitive impairment and dementia: a systematic review and meta-analysis of prospective studies. J Alzheimers Dis. (2024) 98:373–85. doi: 10.3233/JAD-231381 38461506

[135] Dursun E Alaylioglu M Bigic B Hanagasi H Lohmann E Gezen-Ak D . Vitamin D deficiency might pose a greater risk for ApoEϵ4 non-carrier Alzheimer's disease patients. Neurol Sci. (2016) 37:1633–43. doi: 10.1007/s10072-016-2647-1 27357856

[136] Kalmijn S Launer L Ott A Witteman J Hofman A Breteler MB. Dietary fat intake and the risk of incident dementia in the rotterdam study. Neurology. (1997) 42:776–82. doi: 10.1002/ana.410420514 9392577

[137] Barberger-Gateau P Letenneur L Deschamps V Peres K Dartigues JF Renaud S Fish meat and and risk of dementia: cohort study. BMJ. (2002) 325:932–3. doi: 10.1136/bmj.325.7370.932 12399342 PMC130057

[138] Morris M Evans D Bienias J Tagney C Bennett D Schneider J . Consumption of fish and n-3 fatty acids and risk of incident Alzheimer disease. Arch Neurol. (2003) 60:940–6. doi: 10.1001/archneur.60.7.940 12873849

[139] Barberger-Gateau P Raffaitin C Letenneur L Berr C Tzourio C Alpérovitch A . Dietary patterns and risk of dementia: the Three-City cohort study. Neurology. (2007) 69:1921–30. doi: 10.1212/01.wnl.0000278116.37320.52 17998483

[140] Huang T Zandi P Tucker K Fitzpatrick L Kuller L Carlson M . Benefits of fatty fish on dementia risk are stronger for those without APOE epsilon4. Neurology. (2005) 65:1409–14. doi: 10.1212/01.wnl.0000183148.34197.2e 16275829

[141] DacksPA ShinemanDW FillitHM. Current evidence for the clinical use of long-chain polyunsaturated N-3 fatty acids to prevent age-related cognitive decline and Alzheimer's disease. J Nutr Health Aging. (2013) 17:240–51. doi: 10.1007/s12603-012-0431-323459977

[142] Godos J Micek A Currenti W Franchi C Poli A Grosso G . Fish consumption, cognitive impairment and dementia: an updated dose-response meta-analysis of observational studies. Aging Clin Exp Res. (2024) 36:171. doi: 10.1007/s40520-024-02823-6 39162889 PMC11335789

[143] Li L Xu W Tan CC Cao XP Wei BZ Tan L . A gene-environment interplay between omega-3 supplementation and APOE ϵ4 provides insights for Alzheimer's disease precise prevention amongst high-genetic-risk population. Eur J Neurol. (2021) 29:422–31. doi: 10.1111/ene.15160 34710256

[144] Wei BZ Li L Dong CW Tan CC Xu W. The relationship of omega-3 fatty acids with dementia and cognitive decline: evidence from prospective cohort studies of supplementation, dietary intake, and blood markers. Am J Clin Nutr. (2023) 117:1096–109. doi: 10.1016/j.ajcnut.2023.04.001 37028557 PMC10447496

[145] Morris M Evans D Bienias J Tagney C Bennett D Wilson R . Dietary fats and the risk of incident Alzheimer disease. Arch Neurol. (2003) 60:194–200. doi: 10.1001/archneur.60.2.194 12580703

[146] Grant W. A Brief history of the progress in our understanding of genetics and lifestyle, especially diet, in the risk of Alzheimer's disease. J Alzheimers Dis. (2024) 100:S165–78. doi: 10.3233/JAD-240658 39121130 PMC11380269

[147] Barnard N Bunner A Agarwal U. Saturated and trans fats and dementia: a systematic review. Neurobiol Aging. (2014) 35:S65–73. doi: 10.1016/j.neurobiolaging.2014.02.030 24916582

[148] Morris MC Tangney C. Dietary fat composition and dementia risk. Neurobiol Aging. (2014) 35:S59–64. doi: 10.1016/j.neurobiolaging.2014.03.038 24970568 PMC4107296

[149] Liu X Beck T Tangney C Tangney C Desai P Rajan K . Dietary fats and the APOE-e4 risk allele in relation to cognitive decline: a longitudinal investigation in a biracial population sample. J Nutr Health Aging. (2024) 28:100211. doi: 10.1016/j.jnha.2024.100211 38507884 PMC11623058

[150] Cao GY Han L Tayie F Yao SS Huang Z Xu B . Dietary fat intake and cognitive function among older populations: a systematic review and meta-analysis. J Prev Alzheimers Dis. (2019) 6:204–11. doi: 10.14283/jpad.2019.9 31062836

[151] Zhu RZ Chen MQ Zhang ZW Wu TY Zhao WH. Dietary fatty acids and risk for Alzheimer's disease, dementia, and mild cognitive impairment: a prospective cohort meta-analysis. Nutrition. (2021) 90:111355. doi: 10.1016/j.nut.2021.111355 34218119

[152] Saiz-Vazquez O Puente-Martinez A Ubillos-Landa S Pacheco-Bonrostro J Santabarbara J. Cholesterol and Alzheimer's disease risk: a meta-meta-analysis. Brain Sci. (2020) 10:386. doi: 10.3390/brainsci10060386 32570800 PMC7349210

[153] Zhang X Tian Q Liu D Geng T Xu X Wang Y . Causal association of circulating cholesterol levels with dementia: a mendelian randomization meta-analysis. Transl Psychiatry. (2020) 10:145. doi: 10.1038/s41398-020-0822-x 32398686 PMC7217910

[154] Iwagami M Qizilbash N Gregson J Douglas I Johnson M Pocock J . Blood cholesterol and risk of dementia in more than 1.8 million people over two decades: a retropsective cohort study. Lancet Healthy Longev. (2021) 2:e498–506. doi: 10.1016/S2666-7568(21)00150-1 36097999

[155] Wee J Sukudom S Bhat S Marklund M Peiris N Misra A . The relationship between midlife dyslipidemia and lifetime incidence of dementia: a systematic review and meta-analysis of cohort studies. Alzheimers Dement. (2023) 15:e12395. doi: 10.1002/dad2.12395 36911359 PMC9993469

[156] Diaz G Lengele L Sourdet S Soriano G Souto Barreto P. Nutrients and amyloid β status in the brain: a narrative review. Ageing Res Rev. (2022) 81:101728. doi: 10.1016/j.arr.2022.101728 36049590

[157] Yassine H Feng Q Azizkhanian I Rawat V Castor K Chui H . Associated of serum docosahexaenoic acid with cerebral amyloidosis. JAMA Neurol. (2016) 73:1208–16. doi: 10.1001/jamaneurol.2016.1924 27532692

[158] Zhang YP Lou Y Hu J Miao R Ma F. DHA supplementation improves cognitive function via enhancing Aβ-mediated autophagy in Chinese elderly with mild cognitive impairment: a randomised placebo-controlled trial. JAMA Neurol. (2018) 73:1208–16. doi: 10.1136/jnnp-2017-316176 29142143

[159] Jia J Hu J Huo X Miao R Zhang Y Ma F. Effects of vitamin D supplementation on cognitive function and blood Aβ-related biomarkers in older adults with Alzheimer's disease: a randomixed double-blind, blacebo-controlled trial. J Neurol Neurosurg Psychiatry. (2019) 90:1347–52. doi: 10.1136/jnnp-2018-320199 31296588

[160] Kang S Yoo H Cheon B Park Y Kim SJ Seo S . Distinct effects of cholesterol profile components on amyloid and vascular burdens. Alzheimers Res. (2023) 15:197. doi: 10.1186/s13195-023-01342-2 37950256 PMC10636929

[161] Han S Byun M Yi D Jung J Kong N Lee D . Modulatory effect of blood LDL cholesterol on the association between cerebral Aβ and tau deposition in older adults. J Prev Alzheimers Dis. (2024) 11:1767–74. doi: 10.14283/jpad.2024.131 39559888 PMC11573824

[162] Mosconi L Murray J Davies M Williams S Pirraglia E de Leon M . Nutrient intake and brain biomarkers of Alzheimer's disease in at-risk cognitively normal individuals: a cross-sectional neuroimaging pilot study. BMJ Open. (2014) 4:e004850. doi: 10.1136/bmjopen-2014-004850 24961717 PMC4078781

[163] Berti V Murray J Davies M Spector N Tsui W Mosconi L . Nutrient patterns and brain biomarkers of Alzheimer's disease in cognitively normal individuals. J Nutr Health Aging. (2015) 19:413–23. doi: 10.1007/s12603-014-0534-0 25809805 PMC4375781

[164] Kleinert M Clemmensen C Hofmann S Moore M Renner S Tschop M . Animal models of obesity and diabetes mellitus. Nat Rev Endocrinol. (2018) 14:140–62. doi: 10.1038/nrendo.2017.161 29348476

[165] Igura K Ohta T Kuroda Y Kaji K. Resveratrol and quercetin inhibit angiogenesis in vitro. Cancer Lett. (2001) 171:11–6. doi: 10.1016/S0304-3835(01)00443-8 11485823

[166] Yavari M Ramalingam L Harris B Moustaid-Moussa N. Eicosapentaenoic acid protects against metabolic impairments in the APPswe/PS1dE9 Alzheimer's disease mouse model. J Nutr. (2023) 153:1038–51. doi: 10.1016/j.tjnut.2023.01.030 36781072 PMC10273166

[167] Hall K. Predicting metabolic adaptation, body weight change, and energy intake in humans. Am J Physiol Endocrinol Metab. (2009) 298:E449–66. doi: 10.1152/ajpendo.00559.2009 19934407 PMC2838532

[168] Thomas D Ciesla A Levine J Stevens J Martin C. A mathematical model of weight change with adaptation. Math Biosci Eng. (2009) 6:873–87. doi: 10.3934/mbe.2009.6.873 19835433 PMC2764961

[169] Guo J Hall K. Predicting changes of body weight, body fat, energy expenditure and metabolic fuel selection in C57BL/6 mice. PLoS ONE. (2011) 6:e15961. doi: 10.1371/journal.pone.0015961 21246038 PMC3016341

[170] Hall K. Metabolism of mice and men: mathematical modeling of body weight dynamics. Curr Opin Clin Nutr Metab Care. (2012) 15:418–23. doi: 10.1097/MCO.0b013e3283561150 22878236

[171] Hodgkin A Huxley A. A quantitative description of membrane current and its application to conduction and excitation in nerve. J Physiol. (1952) 117:500–44. doi: 10.1113/jphysiol.1952.sp004764 12991237 PMC1392413

[172] McCulloch W Pitts W. A logical calculus of the ideas immanent in nervous activity. Bull Math Biophys. (1943) 5:115–33. doi: 10.1007/BF02478259 2185863

[173] Bertsch M Franchi B Meacci L Primicerio M Tesi MC. The amyloid cascade hypothesis and Alzheimer's disease: a mathematical model. Eur J Appl Math. (2021) 32:749–68. doi: 10.1017/S0956792520000339

[174] Guo L Vardakis J Lassila T Mitolo M Ravikumar N Ventikos Y . Subject-specific multi-poroelastic model for exploring the risk factors associated with the early stages of Alzheimer's disease. Interface Focus. (2017) 8:20170019. doi: 10.1098/rsfs.2017.0019 29285346 PMC5740222

[175] Li Z Chen D Li Z Fan H Guo L Ventikos Y . A computational study of fluid transport characteristics in the brain parenchyma of dementia subtypes. J Biomech. (2023) 159:111803. doi: 10.1016/j.jbiomech.2023.111803 37734184

[176] Mardal KA Rognes ME Thompson TB Valnes LM. Mathematical Modeling of the Human Brain: From Magnetic Resonance Images to Finite Element Simulation. Springer Cham. (2022). doi: 10.1007/978-3-030-95136-8

[177] Weickenmeier J Jucker M Goriely A Kuhl E. A physics-based model explains the prion-like features of neurodegeneration in Alzheimer's disease, Parkinsons's disease, and amyotrophic lateral sclerosis. J Mech Phys Solids. (2019) 124:264–81. doi: 10.1016/j.jmps.2018.10.013

[178] Sporns O. Structure and function of complex brain networks. Dialogues Clin Neurosci. (2013) 15:247–62. doi: 10.31887/DCNS.2013.15.3/osporns 24174898 PMC3811098

[179] Sotiropoulos S Zalesky A. Building connectomes using diffusion MRI: why, how and but. NMR Biomed. (2017) 32:e3752. doi: 10.1002/nbm.3752 28654718 PMC6491971

[180] Tahedl M Tournier JD Smith R. Structural connectome construction using constrained spherical deconvolution in multi-shell diffusion-weighted magnetic resonance imaging. Nat Protoc. (2025) 20:2652–2684. doi: 10.1038/s41596-024-01129-1 39953164

[181] Garyfallidis E Brett M Amirbekian B Rokem A van der Walt S Nimmo-Smith I . DIPY, a library for the analysis of diffusion MRI data. Front Neuroinform. (2014) 8:8. doi: 10.3389/fninf.2014.00008 24600385 PMC3931231

[182] Jenkinson M Beckmann C Behrens T Woolrich M Smith S. FSL. Neuroimage. (2012) 62:782–90. doi: 10.1016/j.neuroimage.2011.09.015 21979382

[183] Tourbier S Rue-Queralt J Glomb K Aleman-Gomez Y Mullier E Hagmann P . Connectome Mapper 3: a flexible and open-source pipeline software for multiscale multimodal human connectome mapping. JOSS. (2022) 7:4248. doi: 10.21105/joss.04248

[184] Tournier JD Smith R Raffelt D Tabbara R Dhollander T Connelly A . MRtrix3: a fast, flexible and open software framework for medical image processing and visualisation. Neuroimage. (2019) 202:116137. doi: 10.1016/j.neuroimage.2019.116137 31473352

[185] Raj A Kuceyeski A Weiner M. A network diffusion model of disease progression in dementia. Neuron. (2012) 73:1204–15. doi: 10.1016/j.neuron.2011.12.040 22445347 PMC3623298

[186] Raj A LoCastro E Kuceyeski A Tosun D Relkin N Weiner M. Network diffusion model of progression predicts longitudinal patters of atrophy and metabolism in Alzheimer's disease. Cell Rep. (2015) 10:359–69. doi: 10.1016/j.celrep.2014.12.034 25600871 PMC5747552

[187] Pandya S Zeighami Y Freeze B Dadar M Collins D Raj A . Predictive model of spread of Parkinson's pathology using network diffusion. Neuroimage. (2019) 192:178–94. doi: 10.1016/j.neuroimage.2019.03.001 30851444 PMC7180066

[188] Pandya S Maia P Freeze B Menke R Talbot K Raj A . Modeling seeding and neuroanatomic spread of pathology in amyotrophic lateral sclerosis. Neuroimage. (2022) 251:118968. doi: 10.1016/j.neuroimage.2022.118968 35143975 PMC10729776

[189] Fornari S Schafer A Jucker M Goriely A Kuhl E. Prion-like spreading of Alzheimer's disease within the brain's connectome. J R Soc Interface. (2019) 16:20190356. doi: 10.1098/rsif.2019.0356 31615329 PMC6833337

[190] Brennan G Goriely A. A network aggregation model for amyloid-β dynamics and treatment of Alzheimer's diseases at the brain scale. J Math Biol. (2025) 90:22. doi: 10.1007/s00285-024-02179-5 39891738 PMC11787187

[191] Thompson TB Meisl G Knowles TPJ Goriely A. The role of clearance mechanisms in the kinetics of pathological protein aggregation involved in neurodegenerative diseases. J Chem Phys. (2021) 154:125101. doi: 10.1063/5.0031650 33810689

[192] Schafer A Mormino E Kuhl E. Network diffusion modeling explains longitudinal tau PET data. Front Neurosci. (2020) 14:566876. doi: 10.3389/fnins.2020.566876 33424532 PMC7785976

[193] Putra P Thompson TB Chaggar P Goriely A. Braiding Braak and Braak: staging patterns and model selection in network neurodegeneraiton. Network Neurosci. (2021) 5:929–56. doi: 10.1162/netn_a_00208 PMC8746141 35024537

[194] Chaggar P Vogel J Binette A Thompson T Strandberg O Goriely A . Regional amyloid load predicts tau and atrophy dynamics. Alzheimers Dement. (2025) 20:e085545. doi: 10.1002/alz.085545

[195] Schafer A Peirlinck KM Linka Kuhl E. Bayesian physics-based modeling of tau propagation in Alzheimer's disease. Front Physciol. (2021) 12:702975. doi: 10.3389/fphys.2021.702975 34335308 PMC8322942

[196] Schafer A Chaggar P Thompson TB Goriely A Kuhl E. Predicting brain atrophy from tau pathology: a summary of clinical findings and their translation into personalized models. Brain Multiphys. (2021) 2:100039. doi: 10.1016/j.brain.2021.100039

[197] Brennan G Thompson TB Oliveri H Rognes ME Goriely A. The role of clearance in neurodegenerative diseases. SIAP. (2023) 84:S172–98. doi: 10.1137/22M1487801

[198] Eigen M. Prionics or the kinetic basis of prion diseases. Biophys Chem. (1996) 63:A1–A18. doi: 10.1016/S0301-4622(96)02250-8 8981746

[199] Thompson TB Chaggar P Kuhl E Goriely A. Protein-protein interactions in neurodegenerative diseases: a conspiracy theory. PLoS Comp Biol. (2020) 16:e1008267. doi: 10.1371/journal.pcbi.1008267 33048932 PMC7584458

[200] Alexandersen C de Haan W Bick C Goriely A. A multi-scale model explains oscillatory slowing and neuronal hyperactivity in Alzheimer's disease. J R Soc Interface. (2023) 20:20220607. doi: 10.1098/rsif.2022.0607 36596460 PMC9810432

[201] Alexandersen C Goriely A Bick C. Neuronal activity induces symmetry breaking in neurodegenerative disease spreading. J Math Biol. (2024) 89:3. doi: 10.1007/s00285-024-02103-x 38740613 PMC11614967

[202] Ahern A Thompson TB Oliveri H Lorthois S Goriely A. Modelling cerebrovascular pathology and the spread of amyloid beta in Alzheimer's disease. Proc R Soc A. (2025) 481:20240548. doi: 10.1098/rspa.2024.0548

[203] ADNI. The Alzheimer's Disease Neuroimaging Initiative. (2025). Available online at: https://adni.loni.usc.edu/ (Accessed May, 2025).

[204] MCSA. The Mayo Clinic Study of Aging. (2025). Available online at: https://www.mayo.edu/research/centers-programs/alzheimers-disease-research-center/research-activities/mayo-clinic-study-aging (Accessed May, 2025).

[205] SBF. The Swedish BioFINDER Study. (2025). Available online at: https://biofinder.se/ (Accessed May, 2025).

[206] UKB. The UK Biobank. (2025). Available online at: https://www.ukbiobank.ac.uk/ (Accessed May, 2025).

[207] Bailey R. Overview of dietary assessment methods for measuring intakes of foods, beverages, and dietary supplements in research studies. Curr Opin Biotechnol. (2022) 70:91–6. doi: 10.1016/j.copbio.2021.02.007 33714006 PMC8338737

[208] Cuparencu C Bulmus-Tüccar T Stranstrup J La Barbera G Roager H Dragstead L. Towards nutrition with precision: unlocking biomarkers as dietary assessment tools. Nat Metab. (2024) 6:1438–53. doi: 10.1038/s42255-024-01067-y 38956322

[209] Jeong SM Lee D Rezende L Giovannucci E. Different correlation of body mass index with body fatness and obesity-related biomarker according to age, sex and race-ethnicity. Sci Rep. (2023) 13:3472. doi: 10.1038/s41598-023-30527-w 36859451 PMC9977890

[210] Aleksandrova K Koelman L Rodrigues C. Dietary patterns and biomarkers of oxidative stress and inflammation: a systematic review of observational and intervention studies. Redox Biol. (2021) 42:101869. doi: 10.1016/j.redox.2021.101869 33541846 PMC8113044

[211] Pollicino F Veronese N Dominguez L Barbagallo M. Mediterranean diet and mitochondria: New findings. Exp Gerontol. (2023) 176:112165. doi: 10.1016/j.exger.2023.112165 37019345

[212] Kullmann S Wagner L Hauffe R Kuhnel A Sandforth L Birkenfeld A . A short-term, high-caloric diet has prolonged effects on brain insulin action in men. Nat Metab. (2025) 7:469–77. doi: 10.1038/s42255-025-01226-9 39984682 PMC11946887

[213] Teixeira S He Y Xu Y Sisley S. Vitamin D enhances insulin sensitivity in neurons. Diabetes. (2018) 67:278-LB. doi: 10.2337/db18-278-LB

[214] Borsini A Nicolaou A Camacho-Munoz D Kendall AC Di Benedetto MG Giacobbe J . Omega-3 polyunsaturated fatty acids protect against inflammation through production of LOX and CYP450 lipid mediators: relevance for major depression and for human hippocampal neurogenesis. Mol Psychiatry. (2021) 26:6773–88. doi: 10.1038/s41380-021-01160-8 34131267 PMC8760043

[215] Dyall S. Long-chain omega-3 fatty acids and the brain: a review of the independent and shared effects of EPA, DPA and DHA. Front Aging Neurosci. (2015) 7:52. doi: 10.3389/fnagi.2015.00052 25954194 PMC4404917

[216] Yassine H Croteau E Rawat V Hibbeln J Rapoport S Umhau J . DHA brain uptake and APOE_4 status: a PET study with [1-^1^1C]-DHA. Alzheimers Res Ther. (2017) 9:23. doi: 10.1186/s13195-017-0250-1 28335828 PMC5364667

[217] Daducci A Gerhard S Griffa A Lemkaddem A Cammoun L Thiran JP . The Connectome Mapper: an open-source processing pipline to map connectomes with MRI. PLoS ONE. (2012) 7:e48121. doi: 10.1371/journal.pone.0048121 23272041 PMC3525592

